# Characterising Distinct Migratory Profiles of Infiltrating T-Cell Subsets in Human Glioblastoma

**DOI:** 10.3389/fimmu.2022.850226

**Published:** 2022-04-06

**Authors:** Paris M. Kollis, Lisa M. Ebert, John Toubia, Cameron R. Bastow, Rebecca J. Ormsby, Santosh I. Poonnoose, Sakthi Lenin, Melinda N. Tea, Stuart M. Pitson, Guillermo A. Gomez, Michael P. Brown, Tessa Gargett

**Affiliations:** ^1^ Translational Oncology Laboratory, Centre for Cancer Biology, SA Pathology and University of South Australia, Adelaide, SA, Australia; ^2^ Adelaide Medical School, Faculty of Health and Medical Sciences, University of Adelaide, Adelaide, SA, Australia; ^3^ Cancer Clinical Trials Unit, Royal Adelaide Hospital, Adelaide, SA, Australia; ^4^ Australian Cancer Research Foundation (ACRF) Cancer Genomics Facility, Centre for Cancer Biology, SA Pathology and University of South Australia, Adelaide, SA, Australia; ^5^ Chemokine Biology Laboratory, Molecular Life Sciences, University of Adelaide, Adelaide, SA, Australia; ^6^ Flinders Health and Medical Research Institute, College of Medicine and Public Health, Flinders University, Adelaide, SA, Australia; ^7^ Department of Neurosurgery, Flinders Medical Centre, Adelaide, SA, Australia; ^8^ Molecular Therapeutics Laboratory, Centre for Cancer Biology, SA Pathology and University of South Australia, Adelaide, SA, Australia; ^9^ Tissue Architecture and Organ Function Laboratory, Centre for Cancer Biology, SA Pathology and University of South Australia, Adelaide, SA, Australia

**Keywords:** T cells, chemokine receptors, integrins, chemokines, glioblastoma, migration, scRNA-seq

## Abstract

Glioblastoma is the most common and aggressive form of primary brain cancer, with no improvements in the 5-year survival rate of 4.6% over the past three decades. T-cell-based immunotherapies such as immune-checkpoint inhibitors and chimeric antigen receptor T-cell therapy have prolonged the survival of patients with other cancers and have undergone early-phase clinical evaluation in glioblastoma patients. However, a major challenge for T-cell-based immunotherapy of glioblastoma and other solid cancers is T-cell infiltration into tumours. This process is mediated by chemokine-chemokine receptor and integrin-adhesion molecule interactions, yet the specific nature of the molecules that may facilitate T-cell homing into glioblastoma are unknown. Here, we have characterised chemokine receptor and integrin expression profiles of endogenous glioblastoma-infiltrating T cells, and the chemokine expression profile of glioblastoma-associated cells, by single-cell RNA-sequencing. Subsequently, chemokine receptors and integrins were validated at the protein level to reveal enrichment of receptors CCR2, CCR5, CXCR3, CXCR4, CXCR6, CD49a, and CD49d in glioblastoma-infiltrating T-cell populations relative to T cells in matched patient peripheral blood. Complementary chemokine ligand expression was then validated in glioblastoma biopsies and glioblastoma-derived primary cell cultures. Together, enriched expression of homing receptor-ligand pairs identified in this study implicate a potential role in mediating T-cell infiltration into glioblastoma. Importantly, our data characterising the migratory receptors on endogenous tumour-infiltrating T cells could be exploited to enhance the tumour-homing properties of future T-cell immunotherapies for glioblastoma.

## Introduction

Glioblastoma is the most common and aggressive form of primary brain cancer in adults (1). Despite standard treatment employing surgery, radiotherapy and chemotherapy, glioblastoma consistently recurs within 6-9 months of initial treatment, illustrating its treatment-resistant nature. Therefore, it is not surprising that the abysmal survival rate of 4.6% at 5 years has not improved over the past three decades (2). With no new or improved survival-prolonging treatment strategies developed in the last 15 years (3), patients and their carers are in desperate need of new therapeutics.

Despite the dramatic success of T-cell-based immunotherapies such as chimeric antigen receptor (CAR) T cells and immune checkpoint inhibitors (ICIs) in patients with other cancers (4, 5), early clinical data indicate that ICIs are only effective in a minority of glioblastoma patients, while CAR T-cell therapy appears promising and safe (6, 7). Among other challenges including immunosuppression and a lack of tumour neoantigens, success of these therapies is limited partly because of restricted T-cell infiltration into glioblastoma (8). The sustained anti-tumour effect of ICIs like anti-PD-1 requires reactivation of endogenous infiltrating anti-tumour T cells (6). Similarly, CAR T cells must be capable of infiltrating tumours to exert their tumour-killing functions (8). In both instances, T cells require the correct migratory receptor repertoire to migrate to and infiltrate the tumour site. Therefore, by enhancing the migratory capabilities of endogenous T cells and CAR T cells alike, T-cell-based immunotherapies could be improved to treat glioblastoma patients.

Despite apparent challenges, such as the blood-brain barrier (BBB) composed of tight-junction endothelial cells (ECs) and astrocyte endfeet (9), some endogenous T cells are nevertheless capable of infiltrating glioblastoma (10). The passage of T cells from the bloodstream into tissues such as tumours is a tightly regulated, multi-step process. This initially involves the tethering and rolling of T cells along the vessel wall, followed by firm adhesion to the endothelium, and finally transmigration through the vessel wall (11). The firm adhesion step is facilitated by chemokine-chemokine receptor axes and integrin-adhesion molecule interactions, whereby chemokines facilitate firm adhesion of T cells to endothelial cells (ECs) *via* integrin activation and binding, then direct the migration of T cells within the tissue parenchyma (12). Only T cells expressing the correct combination of these chemokine and adhesion receptors are expected to effectively infiltrate glioblastoma.

T-cell subsets that participate in anti-tumour immunity, including CD4^+^ and CD8^+^ memory subsets and T regulatory cells (Tregs), have been identified in glioblastoma (10, 13, 14). Based on previous antigen experience, T cells are characterised as naïve, T-central memory (Tcm), T-effector memory (Tem), and T-effector memory re-expressing CD45RA (Temra) populations. Tcm, Tem and Temra differ in their immediate effector function upon antigen exposure and circulation. Tcm cells rapidly respond to secondary antigen exposure and differentiate into effector T-cell populations in secondary lymphoid organs or the site of the response (15). Conversely, Tem and Temra populations recognise antigen directly in tissues and mount immediate effector responses (16). Antigen exposure also gives rise to T-resident memory (Trm) cells, which do not recirculate but reside in peripheral tissues to provide immediate protection against secondary local infections and cancer (17). Recently, Trm cells have been identified in the healthy brain (18) and have been shown to express homing receptors important for tissue retention (19). However, Trm cells have yet to be identified in glioblastoma and the migratory profile of Trm cells in these tumours has not been characterised. The activation- and exhaustion-specific markers of Tregs and activated CD4^+^ and CD8^+^ T cells have been investigated in glioblastoma (14, 20, 21) but the migratory receptors required by these subsets for glioblastoma-homing remain unknown. While T-cell infiltration of glioblastoma is a prerequisite for the efficacy of immunotherapy, a thorough characterisation of the migratory phenotypes on T cells in glioblastoma has not been performed.

Here, using single-cell RNA-sequencing (scRNA-seq) and flow cytometry on glioblastoma patient biopsies, we characterise for the first time the chemokine receptor and integrin expression profiles of endogenous glioblastoma-infiltrating T cells. We identify that T-cell subsets expressing CCR2, CCR5, CXCR3, CXCR4, CXCR6, CD49a, and CD49d are enriched in glioblastoma tumours compared to matched peripheral blood samples. Additionally, validation of complementary chemokines implicates specific chemokine-chemokine receptor axes in T-cell homing and infiltration of glioblastoma tumours. Importantly, these findings identify migratory receptors that could be exploited to improve T-cell-based immunotherapy for glioblastoma and possibly other solid tumours.

## Materials and Methods

### Human Samples

Clinical samples (tumour tissue and blood) from patients with histologically confirmed glioblastoma multiforme were obtained through the South Australian Neurological Tumour Bank (SANTB) and use of the tissues was approved by the Central Adelaide Local Health Network Human Research Ethics Committee (CALHN HREC; approval number R20160727). All human specimens were used in accordance with the Declaration of Helsinki, and participants provided written consent.

### Single Cell Transcriptomics

To interrogate genes related to glioblastoma T-cell homing, we used our previously published scRNA-seq dataset based on a total of 13,903 cells from three primary glioblastoma specimens (22). Refer to Ebert et al. (2020) for detailed methods on tumour specimen processing for single-cell sequencing, as well as single-cell library processing (22). Briefly, using R version 3.6.3 and Seurat version 3.1.5, cells with (i) fewer than 200 genes, (ii) gene numbers outside ±2 standard deviations from the mean, or (iii) a mitochondrial gene fraction greater than 10% were excluded. Remaining cells were log normalised by total expression and scaled to 10,000 transcripts/cell with the NormalizeData function in Seurat. Using the FindIntegrationAnchors (dims = 1:30, k.filter = 200) and IntegrateData (dims = 1:30) functions, cells from different libraries were combined by assessing the pairwise correspondence between a set of representative genes (anchors). The FindVariableGenes function was used to identify variable genes returning 2000 features using vst as the selection method. The data were then scaled and principal component analysis applied using the ScaleData and RunPCA functions respectively. Cells were clustered based on gene expression profiles using the FindNeighbors and FindClusters functions with resolution set to 0.5. UMAP plots were generated using the first 20 principal components, and clusters annotated based on expression of reported cell-lineage markers (23).

To identify different subsets of lymphocytes, the lymphocyte clusters were first isolated from non-lymphocyte clusters. Subsequently, the isolated lymphocyte cells were re-clustered as described above. UMAP plots were generated using the first 11 principal components, and clusters annotated based on expression of cell-lineage markers. *EBF1* and *CD19* delineated B cells and *CD14* and *CD68* identified contaminating macrophages. *CD3D* and *NCR1* identified T-cell and NK-cell-clusters respectively. CD4^+^, CD8^+^ T cells and Tregs were visualised using *CD4*, *CD8* and *FOXP3* respectively. T cells expressing high levels of *TCF7*, *SELL*, *CCR7*, *IL7R*, *PTGER2*, and *KLRG1* were considered Tcm-like. *GZMB*, *PDCD1*, *HLA-DRA*, and *KLRC1* were used to classify effectors or recently activated T cells. Additionally, *ITGA1*, *ITGAE*, and *CXCR6* were used to identify Trm cells (13).

To screen for chemokine receptor, integrin, chemokine, and adhesion molecule gene expression, the frequency of cells positive for genes of interest were obtained using the FetchData function from the merged and normalised libraries of the BT20, BT23 and BT26 scRNA-seq dataset. Cells were considered positive for a gene of interest if at least one unique molecular identifier (UMI) corresponding to the gene was detected in the cell. Cell populations were identified as previously described (22).

### Sample Preparation and Storage

Glioblastoma samples were obtained as pieces of resected tumour tissue, or as tumour fragments recovered from aspirates following Cavitron Ultrasonic Surgical Aspirator (CUSA) ablation, and dissociated as described previously (22). For tumour biopsies, a portion of the sample was embedded in optimal cutting temperature (OCT) medium, flash-frozen using supercooled isopentane, and stored at -80°C. Remaining tumour biopsy was rinsed to remove blood and dissociated to generate single-cell suspensions using Accutase (Thermo Fisher Scientific) or the Human Tumor Dissociation kit in combination with the gentleMACS Octo Dissociator (Miltenyi Biotec) according to the manufacturer’s recommendations. Tissue fragments in CUSA aspirates were first sedimented by slow centrifugation at 70g for 1 min and the supernatant was discarded. The remaining tissue fragments were dissociated using Accutase or the Human Tumor Dissociation kit as described above. Following digestion, dissociated samples were centrifuged at 300g for 5 min to pellet cells, and supernatants for chemokine analyses harvested and stored at -80°C. Dissociated cells were resuspended and filtered through a 70µm strainer. For cryopreservation, a portion of the cells were resuspended in 90% foetal bovine serum (FBS) + 10% dimethyl sulphoxide (DMSO), frozen in a Mr Frosty freezing chamber (Thermo Fisher Scientific) at -80°C overnight, then stored in liquid nitrogen for flow cytometry analyses. The remaining cells were used to generate glioma neural stem (GNS) cell cultures as previously described (22, 24). GNS cell-line supernatants were harvested when the cells were 75-95% confluent and stored at -80°C for chemokine analyses.

Peripheral blood mononuclear cells (PBMC) were harvested from patient blood *via* density centrifugation (Lymphoprep), resuspended in 90% FBS + 10% DMSO, then frozen in a Mr Frosty freezing chamber (Thermo Fisher Scientific) at -80°C overnight and stored in liquid nitrogen for flow cytometry analyses.

### Immunofluorescence Staining of Cryosections

Sections cut from OCT-embedded fresh tissues (5-12µm thickness) were fixed for 20 minutes in Cytofix/Cytoperm (BD) at room temperature (RT), washed in PBS and blocked using 10% human plasma, 10% normal donkey serum and 10% normal goat serum in PBS containing 1% bovine serum albumin (BSA) for 30 minutes at RT. 5µg/mL purified mouse anti-CD3 (UCHT1; BioLegend) and 0.67µg/mL rabbit anti-CD31 (polyclonal; Bethyl Labs #IHC-00055) antibodies were incubated with tissue sections for 12 hours at 4°C. Following washing, tissue sections were incubated with goat anti-mouse IgG-AF488 (Abcam) and donkey anti-rabbit IgG-AF555 (Abcam) detection antibodies at a dilution of 1:500 for 1 hour at RT. Following washing, tissue sections were blocked using 10% normal mouse serum in PBS containing 1% BSA for 30 minutes at RT. Sections were then incubated with mouse anti-GD2-AF647 (14.G2A; BD) at a dilution of 1:100 for 2 hours at RT. After washing, sections were mounted using ProLong Gold anti-fade mounting medium with DAPI (Life Technologies), cured overnight, and sealed with nail varnish before viewing.

### Microscopy and Imaging

Confocal imaging was performed on a Zeiss LSM 800 confocal microscope equipped with 405nm, 488nm, 561nm and 640nm lasers, using 20x objective and Zen 2.6 system software. Fluorescence overlays were created by combining Z-stacks, merging channels, and applying false colour using ImageJ (National Institutes of Health).

### Flow Cytometry

For flow cytometry analysis, cryopreserved, dissociated tumour specimens were rapidly thawed at 37°C, washed, and incubated in pre-warmed Roswell Park Memorial Institute (RPMI) media and 20U/mL DNase I (Sigma) for 15 minutes at 37°C. Cells were then washed and resuspended in a 30% isotonic Percoll (Sigma)/RPMI solution with 2mM EDTA. Using a syringe and needle, 70% isotonic Percoll/RPMI with 2mM EDTA was underlaid beneath the cell suspension, then centrifuged at 500g for 30 mins without the brake at RT. Lymphocytes were harvested from the interphase and washed with complete RPMI media to remove excess Percoll. Isolated lymphocytes were resuspended in PBS and plated at a density of 3x10^5^ cells/well in 96-well V-bottom plates. Cryopreserved patient PBMC were rapidly thawed at 37°C, washed, and resuspended in PBS to be plated at a density of 5x10^5^ cells/well. Five flow cytometry panels were designed to simultaneously analyse chemokine receptor and/or integrin expression while identifying T-cell subsets. A core combination of antibodies to identify T-cell subsets including: anti-CD3-PerCP-Cy5.5 (UCHT1; BD), anti-CD4-BV510 (SK3; BD), anti-CD8-BV786 (RPA-T8; BD), anti-CD25-BB515 (2A3; BD), anti-CD69-PE-Cy7 (FN50; BD), anti-CD45RO-BUV395 (UCHL1; BD), anti-CCR7-APC (2-L1-A; BD) or anti-CCR7-BV421 (G043H7; BD), anti-CD127-PE (A019D5; Biolegend), and anti-CD103-BV605 (Ber-ACT8; Biolegend) were used in each panel with an additional 2-3 antibodies to detect different chemokine receptors and/or integrins. Patient samples were randomly allocated to the different flow panels with larger biopsies that provided a greater yield of T cells used in multiple flow panels. Patient tumour samples digested with Accutase were allocated to the flow cytometry panel containing CXCR3 and CCR6 antibodies as the standard dissociation protocol with GentleMACS reagents (Miltenyi) significantly reduced the detection of these receptors. Cells were pelleted *via* centrifugation at 400g for 3 minutes, resuspended in PBS with 1:1000 Fixable viability stain 780 (BD) and incubated for 20 minutes at RT. Cells were washed with FACS buffer (PBS, 1% BSA) and Fc receptors blocked with human Fc block (BD) for 5 minutes on ice. For chemokine receptor analyses, the following antibodies were used: anti-CCR2-BUV737 (LS132.1D9, BD), anti-CCR4-BV421 (1G1; BD), anti-CCR5-PE-CF594 (2D7; BD), anti-CCR6-BUV737 (11A9; BD), anti-CCR7-APC, anti-CCR7-BV421, anti-CXCR3-APC (G025H7; Biolegend), anti-CXCR4-BV421 (12G5; BD), anti-CXCR6-BV421 (13B 1E5; BD), and anti-CX3CR1-PE-CF594 (2A9-1; BD). For panels with anti-CCR5, chemokine receptor staining was staggered as previously recommended (25). First, cells were incubated with chemokine receptor antibodies, except anti-CCR5, in Brilliant Staining Buffer Plus (BSB+; BD) for 10 mins at RT. Anti-CCR5-PE-CF594 was then added and cells were incubated for a further 10 minutes at RT. For panels without anti-CCR5, cells were incubated with chemokine receptor antibodies in BSB+ for 20 mins at RT. To the above suspensions, our core T-cell antibody cocktail diluted in BSB+ (BD) was added and incubated for a further 20 minutes on ice. For integrin panels, cells were incubated with anti-CCR7 at RT for 20 minutes, then incubated for a further 20-minutes on ice with the above T-cell cocktail supplemented with anti-CD11a-BV421 (HI111; BD) and anti-CD49d-BUV737 (9F10; BD), or anti-CD51-APC (NKI-M9; Biolegend) and anti-CD49a-BUV737 (SR84; BD). Stained cells were washed with PBS and fixed with Cytofix/Cytoperm (BD) for 20 minutes on ice. Lastly, cells were washed with PBS, resuspended in FACS buffer, and stored at 4°C protected from light until acquisition. Samples were acquired on a BD LSR Fortessa Special Order Research Product using FACS Diva Software version 8.0. Analysis was performed using FlowJo™ version 10.8.1 (BD).

### Enzyme-Linked Immunosorbent Assay (ELISA)

To detect CXCL16 and CCL20 in supernatants harvested from GNS cell-lines and dissociated glioblastoma and CUSA samples, the DuoSet ELISA Human CXCL16 kit (DY1164; R&D Systems) and DuoSet ELISA Human CCL20 kit (DY36005; R&D Systems) were used according to the manufacturer’s recommendations. Samples were analysed at 450 nm (FLUOstar Omega microplate reader), and concentrations of chemokines calculated from standard curves generated with GraphPad Prism version 8.4.3.

### Cytometric Bead-Based Immunoassay

To detect the chemokines CCL2, CCL3, CCL4, CCL5, CXCL9, CXCL10, CXCL11, CXCL12 and CX3CL1 in supernatants harvested from GNS cell-lines and dissociated glioblastoma and CUSA samples, a LEGENDplex Custom Human Chemokine Panel kit (Biolegend) was used according to the manufacturer’s recommendations. Standards and samples were acquired on a BD LSR Fortessa Special Order Research Product and chemokine concentrations determined using the LEGENDplex data analysis software version 2021-07-01 (Biolegend).

### Statistical Analyses

Statistical analysis and graphing were performed in GraphPad Prism version 8.4.3. Pairwise comparisons were performed using two-tailed paired Student’s t test. For all tests, p values under 0.05 were considered significant. Significance was represented as: *p<0.05, **p<0.01, ***p<0.001, ****p<0.0001.

## Results

### Endogenous T Cells Infiltrate Glioblastoma

**Figure 1:**
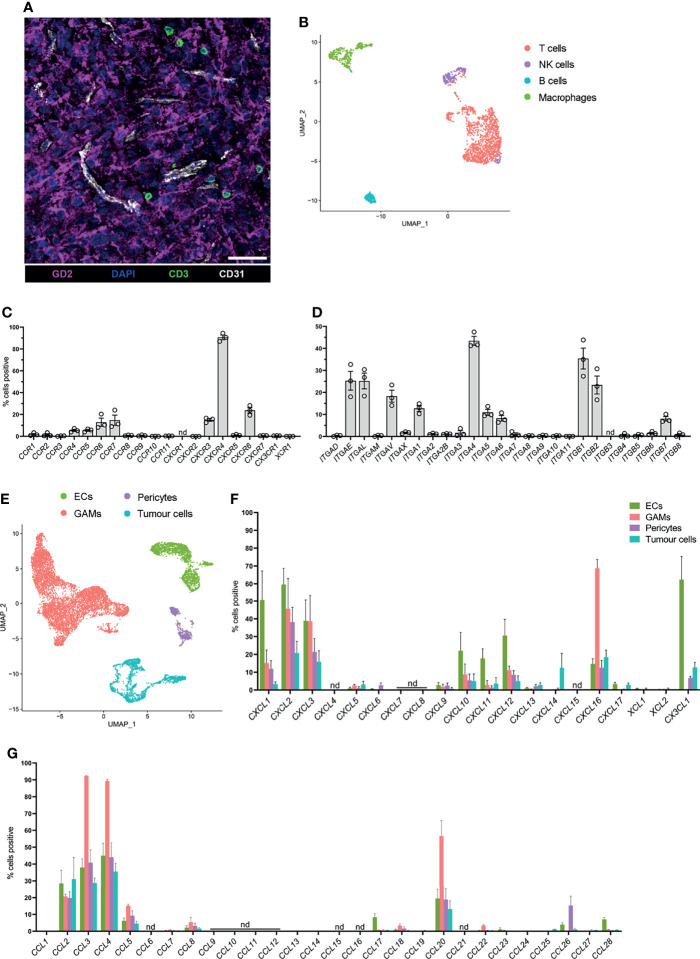
Endogenous T cells that infiltrate glioblastoma express distinct homing marker genes, and complementary chemokine genes are detected in other glioblastoma-associated cell clusters. **(A)** Representative 7μm glioblastoma tissue section identifying endogenous CD3^+^ T cells (green) localised adjacent to tumour-associated CD31^+^ blood endothelial cells (white) and infiltrating GD2^+^ tumour parenchyma (magenta). DAPI was used as a nuclear stain, scale bar: 50µm, representative of n=6 patient samples. **(B)** UMAP plot of T cells, NK cells, B cells, and macrophages identified within the lymphocyte cluster from pooled scRNA-seq analysis of n=3 patient glioblastoma biopsies. Cell clusters were identified according to differentially expressed genes (see **Supplementary Figure 1**). **(C, D)** Frequency of T cells within the lymphocyte cluster positive for **(C)** chemokine receptor and **(D)** integrin genes. Each dot represents one patient, bars represent mean ± SEM. nd=not detected. **(E)** UMAP plot of non-lymphocyte glioblastoma-associated cell clusters from pooled scRNA-seq analysis of n=3 patient glioblastoma biopsies. Endothelial cells (ECs), glioblastoma-associated macrophages and microglia (GAMs), pericytes and tumour cells were clustered and identified according to differentially expressed genes. **(F, G)** Frequency of ECs, GAMs, pericytes and tumour cells positive for **(F)** *CXCL*-, *XCL*- and *CX3CL1*, and **(G)** *CCL*-chemokine genes. Bars represent mean ± SEM, nd, not detected.

To identify glioblastoma tissues with infiltrating T cells, frozen sections were prepared from patients’ biopsies (n=6) for confocal microscopy. Sections were stained for CD3 (green) to identify glioblastoma-infiltrating T cells, GD2 (magenta) to visualise tumour cells, and CD31 (white) to identify ECs (**Figure 1A**). In all instances, T cells were visualised near CD31^+^ blood ECs and among GD2^+^ glioblastoma cells (**Figure 1A**), but not within blood vessels. Importantly, this confirmed that glioblastoma tissues contained endogenous T cells that had infiltrated the glioblastoma parenchyma and were not restricted to the blood vessel lumen.

Three of these glioblastoma biopsies, all of which contained tumour-infiltrating T cells, (BT20, BT23, BT26; refer to **Table 1**), were matched to our previously published scRNA-seq dataset (22). This dataset was generated by dissociating three primary glioblastoma patient specimens (**Table 1**) to single-cell suspensions and subjecting them to scRNA-seq using the 10X Chromium platform. Detailed methods and data analysis pipelines are described in the materials and methods section and our recent publication (22). Upon clustering of cells based on their transcriptome, expression of reported lineage-markers was used to identify the cell-type of each cluster. Subsequently, clusters were identified as ECs, glioblastoma-associated macrophages/microglia (GAMs), pericytes, and lymphocytes (**Supplementary Figure 1A**). Clustered cells expressing a range of genes associated with glial and neural cell types but lacking stromal lineage-markers were annotated as tumour cells (**Supplementary Figure 1A**). Utilising this existing dataset, expression of chemokine receptors and integrins was interrogated in glioblastoma-infiltrating T cells for this study.

**Table 1:** Glioblastoma patient demographics and their samples used in this study.

Patient ID	Sex	Age	Diagnosis	IDH mutation status	Samples available	Use in study
BT4	Female	59	Primary glioblastoma	Not reported in patient notes	Tumour biopsy	Flow cytometry
					Blood	Flow cytometry
BT8	Female	49	Recurrent glioblastoma	Not reported in patient notes	CUSA	Flow cytometry
					Blood	Flow cytometry
BT15	Male	74	Recurrent glioblastoma	Not reported in patient notes	Tumour biopsy	Flow cytometry
					Blood	Flow cytometry
BT16	Female	58	Glioblastoma	IDH WT	CUSA	Flow cytometry
					Blood	Flow cytometry
BT18	Female	33	Recurrent glioblastoma	Not reported in patient notes	CUSA	Flow cytometry
					Blood	Flow cytometry
BT20	Male	55	Primary glioblastoma	Not reported in patient notes	Tumour biopsy	scRNA-seq IF Flow cytometry
					CUSA	Flow cytometry
					Blood	Flow cytometry
					GNS cell-line	ELISA
BT23	Male	78	Primary glioblastoma	Not reported in patient notes	Tumour biopsy	IF
					CUSA	scRNA-seq Flow cytometry
					Blood	Flow cytometry
BT26	Male	69	Primary glioblastoma	Not reported in patient notes	Tumour biopsy	IF
					CUSA	scRNA-seq Flow cytometry
					Blood	Flow cytometry
					GNS cell-line	Chemokine quantification
BT29	Male	66	Glioblastoma	IDH WT	GNS cell-line	Chemokine quantification
BT38	Male	78	Glioblastoma	Not reported in patient notes	Tumour biopsy	Flow cytometry
					Blood	Flow cytometry
					GNS cell-line	Chemokine quantification
BT39	Male	65	Glioblastoma	IDH WT	GNS cell-line	Chemokine quantification
BT41	Male	67	Glioblastoma	IDH WT	GNS cell-line	Chemokine quantification
BT43	Male	45	Glioblastoma	IDH WT	Tumour biopsy	Flow cytometry
					Blood	Flow cytometry
BT45	Male	49	Glioblastoma	Not reported in patient notes	Tumour biopsy	Flow cytometry
					Blood	Flow cytometry
BT48	Male	68	Glioblastoma	Not reported in patient notes	Tumour biopsy	Flow cytometry
					Blood	Flow cytometry
BT49	Female	50	Glioblastoma	Not reported in patient notes	Tumour biopsy	Flow cytometry IF
					Blood	Flow cytometry
BT50	Female	67	Glioblastoma	Not reported in patient notes	Tumour biopsy	Chemokine quantification Flow cytometry IF
					Blood	Flow cytometry
BT52	Female	63	Glioblastoma	IDH1 WT	Tumour biopsy	Flow cytometry
					Blood	Flow cytometry
BT53	Male	67	Glioblastoma	Not reported in patient notes	Tumour biopsy	Flow cytometry
					Blood	Flow cytometry
					GNS cell-line	Chemokine quantification
BT55	Female	78	Glioblastoma	IDH WT	GNS cell-line	Chemokine quantification
BT57	Female	68	Glioblastoma	IDH WT	Tumour biopsy	Chemokine quantification Flow cytometry
					Blood	Flow cytometry
BT58	Male	69	Primary glioblastoma	IDH WT	Tumour biopsy	Chemokine quantification Flow cytometry
					CUSA	Chemokine quantification
					Blood	Flow cytometry
					GNS cell-line	Chemokine quantification
BT59	Male	62	Glioblastoma	Not reported in patient notes	Tumour biopsy	Chemokine quantification Flow cytometry
					CUSA tumour	Chemokine quantification
					Blood	Flow cytometry
					GNS cell-line	Chemokine quantification
BT61	Female	42	Secondary glioblastoma arising from pre-existing grade 2 astrocytoma	Not reported in patient notes	Tumour biopsy	Chemokine quantification
BT62	Female	53	Likely glioblastoma but may be anaplastic oligodendroglioma	IDH1 R132H negative	Tumour biopsy	Chemokine quantification
					CUSA	Chemokine quantification
BT63	Female	36	Recurrent glioblastoma	Not reported in patient notes	Tumour biopsy	Chemokine quantification
					GNS cell-line	Chemokine quantification
BT64	Female	64	Glioblastoma	IDH WT	Tumour biopsy	Chemokine quantification Flow cytometry
					CUSA	Chemokine quantification
					Blood	Flow cytometry
BT66	Male	32	Glioblastoma	Not reported in patient notes	Tumour biopsy	Flow cytometry
					Blood	Flow cytometry
					GNS cell-line	Chemokine quantification
BT67	Female	64	Recurrent glioblastoma	Not reported in patient notes	Tumour biopsy	Chemokine quantification
BT68	Female	68	Glioblastoma	IDH WT	Tumour biopsy	Chemokine quantification

CUSA, Cavitron Ultrasonic Surgical Aspirator; GNS, glioma neural stem; IDH, isocitrate dehydrogenase; IF, immunofluorescence; scRNA-seq, single-cell RNA-sequencing; WT, wild-type.

### Glioblastoma-Associated T Cells Express Chemokine Receptor and Integrin Genes

To screen for chemokine receptor and integrin genes expressed by glioblastoma-infiltrating T cells, the lymphocyte cluster was isolated and re-clustered with the bioinformatics pipeline described (see above and Materials and Methods) to further separate lymphocyte populations according to differentially expressed genes (**Figure 1B**). B cells were identified *via EBF1* and *CD19* expression, contaminating macrophages *via CD14* and *CD68* expression, T cells *via CD3D* expression and NK cells *via NCR1* expression (**Figure 1B** and **Supplementary Figures 1B, C**). Frequencies of T cells positive for chemokine receptor (**Figure 1C**) and integrin genes (**Figure 1D**) in each patient were determined. Within glioblastoma-infiltrating T cells, a large proportion of cells were positive for *CXCR4* and the proportion of cells positive for chemokine receptors *CCR4*, *CCR5*, *CCR6*, *CCR7*, *CXCR3*, and *CXCR6* ranged between 5-25% (**Figure 1C**). Additionally, integrin genes *ITGAE*, *ITGAL*, *ITGAV*, *ITGA1*, *ITGA4*, *ITGA5*, *ITGA6*, *ITGB1*, *ITGB2*, and *ITGB7* were detected in a moderate proportion of T cells (**Figure 1D**). All other receptors investigated were either undetectable or were expressed in low frequencies of T cells. Remaining cells in the lymphocyte cluster were also screened for chemokine receptor and integrin gene expression. B cells, NK cells and contaminating macrophages also expressed chemokine receptor and integrin genes with some overlap in the expression of receptors with T cells (**Supplementary Figures 2A, B**). Together, these data identify infiltrating T cells in glioblastoma biopsies and define a putative expression profile of glioblastoma-homing markers.

### Glioblastoma-Associated Cell Populations Express Complementary Chemokine Genes

Chemokine receptor-mediated T-cell recruitment is directed by complementary chemokine ligands expressed in the glioblastoma microenvironment; thus, the expression of chemokines was screened in non-lymphocyte clusters (**Figure 1E**). There were varying proportions of cells positive for chemokines *CCL2*, *CCL3*, *CCL4*, *CCL5*, *CCL20*, *CXCL1*, *CXCL2*, *CXCL3*, *CXCL10*, *CXCL11*, *CXCL12*, *CXCL16*, and *CX3CL1* (**Figures 1F, G**), among non-lymphocyte cell populations. Subsequently, chemokine receptors identified in glioblastoma-infiltrating T cells were selected for further investigation based on expression of the receptor at the gene level within T-cell clusters, and/or expression of the cognate ligand(s) identified within non-lymphocyte clusters. Thus, the chemokine receptors CCR2, CCR4, CCR5, CCR6, CXCR3, CXCR4, CXCR6, and CX3CR1 were selected for further validation by flow cytometry. Similarly, integrins CD49a (*ITGA1*), CD49d (*ITGA4*), CD51 (*ITGAV*), and CD11a (*ITGAL*) were selected for further validation based on elevated gene expression in glioblastoma-infiltrating T cells.

### Analysis of T-Cell Subsets in Glioblastoma Biopsies and Matched Blood Samples

**Figure 2:**
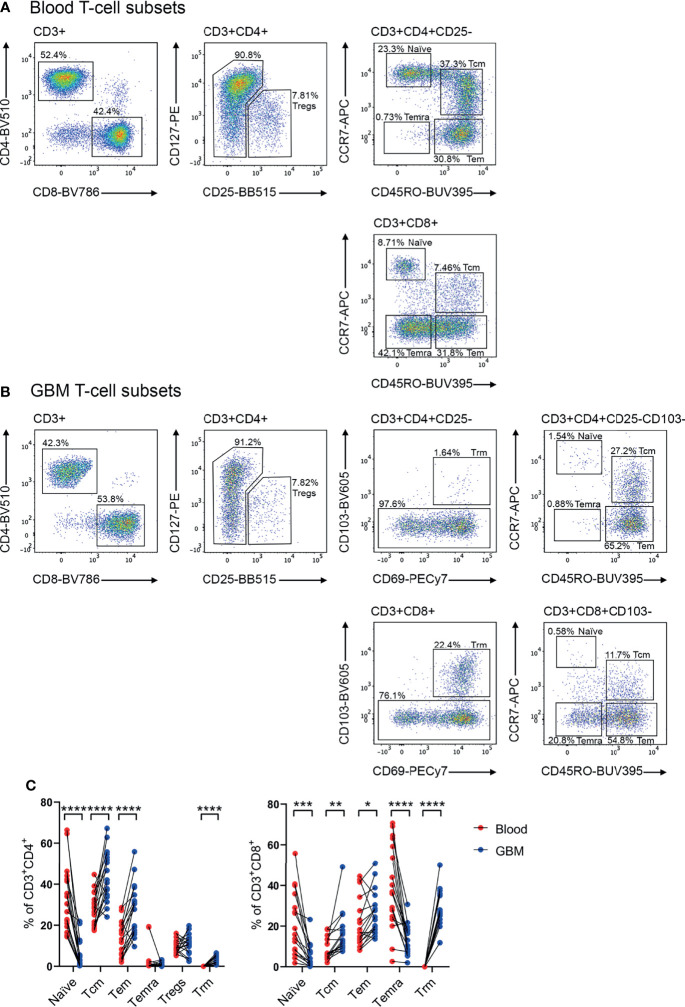
Identification of T-cell subsets in glioblastoma-patient blood and tumour biopsies. Representative flow cytometry gating strategy for **(A)** blood- and **(B)** glioblastoma (GBM)- associated T cells. Staining of lineage-specific surface markers identifies T regulatory cells (Tregs: CD3^+^CD4^+^CD25^+^CD127^low^), and CD4^+^ and CD8^+^ naïve T cells (CD103^-^CD45RO^-^CCR7^+^), T central memory (Tcm: CD103^-^CD45RO^+^CCR7^+^), T effector memory (Tem: CD103^-^CD45RO^+^CCR7^-^), T effector memory re-expressing CD45RA (Temra: CD103^-^CD45RO^-^CCR7^−^), and T resident memory (Trm: CD103^+^CD69^+^) populations. Plots are representative of matched patient blood and GBM samples n=18 each. **(C)** Proportions of naïve, Tcm, Tem, Temra, Tregs and Trm cell subsets in GBM patient blood and dissociated tumour biopsies. Proportions are presented as frequency of CD3^+^CD4^+^ and CD3^+^CD8^+^ T-cell populations, respectively. Each dot represents one patient, lines represent paired patient samples. Two-tailed paired Student’s t test, *p < 0.05, **p < 0.01, ***p < 0.001, ****p < 0.0001.

Previous studies have identified that memory T cells, including Tcm, Tem and Temra, and CD4^+^ Tregs can infiltrate glioblastoma (10, 13, 14, 20). Additionally, Trm cells have been characterised in the healthy brain (18). In keeping with this, our scRNA-seq data revealed heterogenous expression of genes associated with a Tcm-like signature (*TCF7*, *SELL*, *CCR7*, *IL7R*, *PTGER2*, and *KLRG1*), and effector-like genes (*GZMB*, *PDCD1*, *HLA-DRA*, and *KLRC1*) (**Supplementary Figure 1C**) (13). Additionally, our scRNA-seq dataset revealed *FOXP3* expression in a small proportion of glioblastoma Tregs, and expression of *ITGA1*, *ITGAE*, and *CXCR6* which are associated with a Trm signature (**Supplementary Figure 1C**) (13). Considering these subsets play distinct roles in tumour immunity (10, 17, 26), a multicolour flow cytometry panel was designed to simultaneously identify these populations and interrogate chemokine receptor and integrin expression (**Figures 2A, B**). Paired blood (**Figure 2A**) and dissociated glioblastoma samples (**Figure 2B**) were analysed to identify homing receptors enriched on glioblastoma-infiltrating T-cell subsets compared to paired blood samples as a means of identifying migratory axes that may be of biological importance. Tregs were identified as CD4^+^CD25^+^CD127^low^ (27), and CD4^+^ or CD8^+^ Trm cells as CD69^+^CD103^+^ (18). Trm cells were not detected in the peripheral blood samples and only identified within glioblastoma. Within the CD103^-^ population, CCR7 and CD45RO expression patterns distinguished naïve (CCR7^+^CD45RO^-^), Tcm (CCR7^+^CD45RO^+^), Tem (CCR7^-^CD45RO^+^), and Temra (CCR7^-^CD45RO^-^) populations for both CD4^+^ and CD8^+^ T cells (10, 16). Following identification of T-cell subsets, frequencies of these subsets were compared between matched patient blood and glioblastoma biopsies (**Figure 2C**). Of note, significantly fewer naïve CD4^+^ and CD8^+^ T cells were detected in glioblastoma biopsies compared to paired blood samples, indicating limited blood contamination of these resected glioblastoma biopsies obtained at surgery. In contrast, there was a significant enrichment of CD4^+^ and CD8^+^ Tcm, Tem, and Trm populations in glioblastoma biopsies, with CD4^+^ Tcm and CD8^+^ Tem and Trm populations most abundant overall. There was also a significant decrease in the proportion of CD8^+^ Temra cells in glioblastoma compared to blood samples, while the proportions of CD4^+^ Temra cells in blood and glioblastoma were negligible. Proportions of Tregs between the blood and tumour samples were similar.

### CCR2^+^, CCR5^+^, CXCR3^+^ and CXCR6^+^ CD4^+^ T-Cell Subsets Are Enriched in Glioblastoma

**Figure 3:**
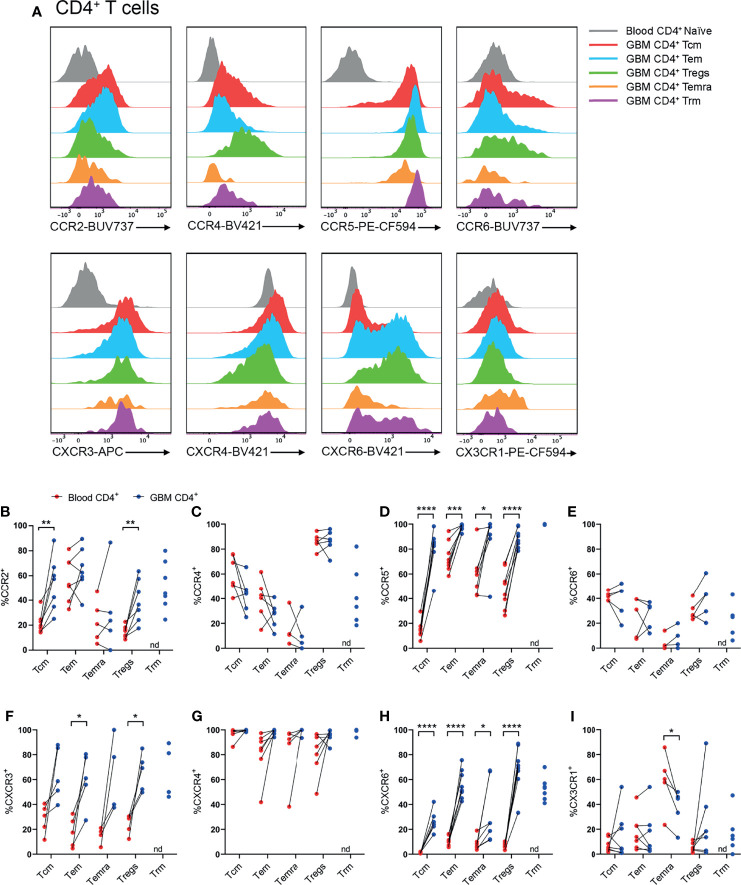
CCR2^+^, CCR5^+^, CXCR3^+^ and CXCR6^+^ CD4^+^ T-cell populations are significantly enriched in patient glioblastoma compared to peripheral blood. **(A)** Representative histograms showing chemokine receptor expression within CD4^+^ T-cell subsets in glioblastoma (GBM) (coloured) and naïve CD4^+^ T cells in matched blood samples (grey). Also see **Supplementary Figure 4** for additional blood CD4^+^ T-cell subsets. Representative of matched blood and GBM samples n=5-8 each. **(B–I)** Proportions of CD4^+^ T-cell subsets in blood and matched GBM biopsies expressing **(B)** CCR2, **(C)** CCR4, **(D)** CCR5, **(E)** CCR6, **(F)** CXCR3, **(G)** CXCR4, **(H)** CXCR6 and **(I)** CX3CR1. Each dot represents one patient, lines represent paired patient sample. Two-tailed paired Student’s t test, *p < 0.05, **p < 0.01, ***p < 0.001, ****p < 0.0001, nd=not detected.

**Figure 4:**
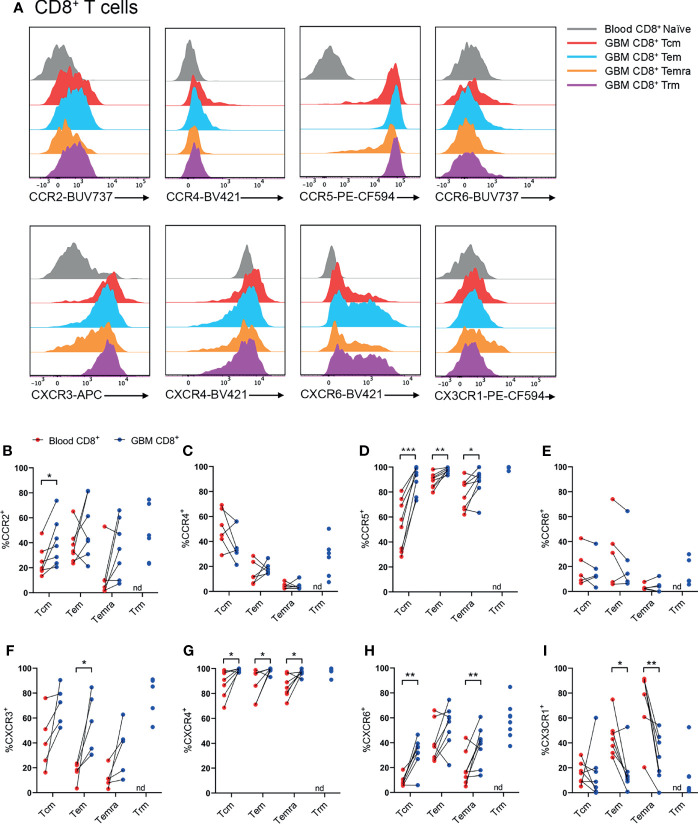
CCR2^+^, CCR5^+^, CXCR3^+^, CXCR4^+^ and CXCR6^+^ CD8^+^ T-cell populations are significantly enriched in patient glioblastoma compared to peripheral blood. **(A)** Representative histograms showing chemokine receptor expression within CD8^+^ T-cell subsets in glioblastoma (GBM) (coloured) and naïve CD8^+^ T cells in matched blood samples (grey). Also see **Supplementary Figure 6** for additional blood CD8^+^ T-cell subsets Representative of n=5-8 matched blood and GBM samples. **(B–I)** Proportions of CD8^+^ T-cell subsets in blood and matched GBM biopsies expressing **(B)** CCR2, **(C)** CCR4, **(D)** CCR5, **(E)** CCR6, **(F)** CXCR3, **(G)** CXCR4, **(H)** CXCR6 and **(I)** CX3CR1. Each dot represents one patient, lines represent paired patient sample. Two-tailed paired Student’s t test, *p < 0.05, **p < 0.01, ***p < 0.001, nd, not detected.

Following identification of T-cell subsets (**Figure 2**), expression of CCR2, CCR4, CCR5, CCR6, CXCR3, CXCR4, CXCR6, and CX3CR1 was investigated on glioblastoma and blood CD4^+^ (**Figure 3**) and CD8^+^ (**Figure 4**) T-cell subsets. Here, naïve blood T cells served as an internal negative control for chemokine receptor staining as this population lacks the expression of inflammatory chemokine receptors, except for CXCR3 and CXCR4, where naïve T cells and live CD3^-^ cells containing distinct negative and positive populations were used to set gates for these receptors respectively (**Supplementary Figure 3**).

Our standard process for dissociation of tumour tissue specimens using gentleMACS reagents was found to significantly diminish CXCR3 and CCR6 staining (data not shown). Hence, other patient samples digested with Accutase were used to interrogate these receptors. Representative histograms for CD4^+^ T-cell subsets illustrate the typical chemokine receptor expression patterns observed, and demonstrate that staining for CCR2, CCR5, CXCR3 and CXCR6 was robust in glioblastoma-infiltrating T-cell populations compared to matched blood naïve CD4^+^ T cells (**Figure 3A** and **Supplementary Figure 4**). Quantitative analysis of chemokine receptor expression revealed a significant increase in the proportion of CCR2^+^CD4^+^ Tcm and Treg cells in glioblastoma compared to blood samples from the same patients (**Figure 3B**). CCR4 expression was most abundant in Tregs, however there was no difference in the proportion of CCR4^+^ Tregs in glioblastoma compared to peripheral blood (**Figure 3C**). Strikingly, all glioblastoma CD4^+^ T-cell subsets highly expressed CCR5, and CCR5 expression was significantly enriched in glioblastoma T cells compared to blood (**Figure 3D**). CCR6 expression was relatively low in CD4^+^ T-cell subsets compared to other chemokine receptors analysed, and no significant differences in CCR6 expression were observed between T-cell subsets in glioblastoma and blood (**Figure 3E**). CXCR3^+^ cells were abundant in all CD4^+^ T-cell subsets, however enrichment was only statistically significant in CD4^+^ Tem and Tregs in glioblastoma biopsies compared to paired blood samples (**Figure 3F**). Equally high proportions of CD4^+^ T-cell subsets in both blood and glioblastoma expressed CXCR4, hence no enrichment of this receptor was observed in glioblastoma (**Figure 3G**). As high proportions of both blood and glioblastoma-associated CD4^+^ T-cell subsets expressed CXCR4, the geometric mean fluorescence intensity (gMFI) was compared to determine whether CXCR4 abundance on T-cell subsets differed between glioblastoma and blood samples. Strikingly, there was a significant increase in the abundance of CXCR4 on all CD4^+^ T-cell subsets in glioblastoma compared to blood samples (**Supplementary Figure 5A**). CXCR6, an established marker of CD69^+^CD103^+^ Trm cells, was expressed on CD4^+^ Trm cells as anticipated, but also enriched in all other CD4^+^ T-cell subsets in glioblastoma compared to blood (**Figure 3H**). Lastly, CX3CR1 expression was low among all CD4^+^ T-cell subsets except CD4^+^ Temra cells, where CX3CR1 expression was enriched in the blood and significantly lower in glioblastoma (**Figure 3I**). Together, our flow cytometry analysis demonstrates that CCR5 and CXCR6 are expressed by large proportions of all glioblastoma-infiltrating CD4^+^ T-cell subsets, whereas CCR2 and CXCR3 enrichment was only observed in glioblastoma-infiltrating CD4^+^ Tcm and Tregs, and Tem and Tregs respectively.

### CCR2^+^, CCR5^+^, CXCR3^+^, CXCR4^+^ and CXCR6^+^ CD8^+^ T-Cell Subsets Are Enriched in Glioblastoma

Within CD8^+^ T-cell subsets, representative histograms highlighted robust expression of CCR5, CXCR3, and CXCR6 compared to naïve peripheral blood CD8^+^ T cells (**Figure 4A** and **Supplementary Figure 6**). Similar to CD4^+^ T cells, there was enrichment of CCR2^+^CD8^+^ Tcm cells in glioblastoma compared to paired blood samples, and approximately 50% of CD8^+^ glioblastoma Trms expressed CCR2 (**Figure 4B**). CCR4^+^ cells were most abundant within the CD8^+^ Tcm population, but expression of this receptor in glioblastoma samples was not enriched for any CD8^+^ T-cell subsets compared to matched blood samples (**Figure 4C**). Like CD4^+^ T-cell subsets, CCR5 was expressed by a high proportion of all CD8^+^ T-cell subsets and was significantly enriched for all CD8^+^ T-cell subsets in glioblastoma (**Figure 4D**). CCR6 was expressed at similar proportions between blood and glioblastoma specimens for all CD8^+^ T-cell subsets (**Figure 4E**). Like CD4^+^ glioblastoma T cells, CXCR3 was expressed moderately by all CD8^+^ T-cell subsets, and there was a significantly greater proportion of CXCR3^+^CD8^+^ Tem cells in glioblastoma biopsies compared to paired blood samples (**Figure 4F**). CXCR4 expression was uniformly high for all CD8^+^ T-cell subsets and significantly enriched in glioblastoma compared to peripheral blood (**Figure 4G**). Additionally, CXCR4 gMFI was significantly increased for all CD8^+^ T-cell subsets in glioblastoma (**Supplementary Figure 5B**). Although all CD8^+^ glioblastoma T-cell subsets expressed modest levels of CXCR6, expression was only enriched within glioblastoma compared to blood for Tcm and Temra cells (**Figure 4H**). Lastly, CX3CR1 expression was greatest in the blood for CD8^+^ Tem and Temra populations with a significant reduction in the frequency of CX3CR1^+^CD8^+^ Tem and Temra cells in glioblastoma samples. Overall, CCR5 and CXCR4 show the most prominent expression on CD8^+^ T-cell subsets in glioblastoma tissues, with CCR2, CXCR3 and CXCR6 displaying subset-specific glioblastoma enrichment in Tcm, Tem, and Tcm and Temra populations respectively.

### CD11a^+^, CD49a^+^ and CD49d^+^ T Cells Are Abundant and/or Enriched in Glioblastoma

**Figure 5:**
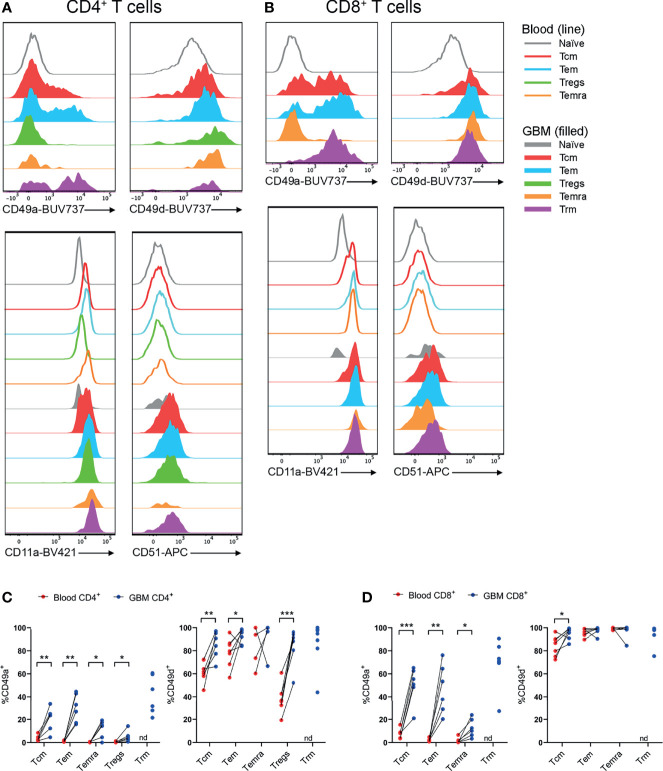
CD49a^+^ and CD49d^+^ T cells are enriched in glioblastoma biopsies compared to peripheral blood. **(A, B)** Representative histograms showing CD49a, CD49d, CD11a and CD51 expression within **(A)** CD4^+^ and **(B)** CD8^+^ T-cell subsets in glioblastoma (GBM) biopsies (filled) and T cells in matched blood samples (line). Also see **Supplementary Figures 4**, **6** for additional blood T-cell subsets Representative of matched blood and GBM samples n=6-7 each. **(C, D)** Proportions of CD49a- and CD49d-expressing **(C)** CD4^+^ and **(D)** CD8^+^ T-cell subsets in blood and matched GBM biopsies. Each dot represents one patient, lines represent paired patient samples. Two-tailed paired Student’s t test, *p < 0.05, **p < 0.01, ***p < 0.001, nd, not detected.

Utilising the same core panel for identification of T-cell subsets as **Figures 3**, **4**, integrins identified by scRNA-seq were investigated by flow cytometry. Subsequently, proportions of T cells expressing integrins were compared between paired blood and glioblastoma samples (**Figure 5**). All T-cell subsets in blood and glioblastoma biopsies uniformly expressed high levels of CD11a (integrin αL; **Figures 5A, B** and **Supplementary Figures 4**, **6**). Positive gates for CD49a and CD51 expression on glioblastoma T-cell subsets were set based on paired blood naïve T cells, which were uniformly CD49a^-^ and CD51^-^ (**Figures 5A, B** and **Supplementary Figure 3**). Live CD3^-^ cells containing distinct populations of CD49d^+^ and CD49d^-^ cells were used to set positive gates for CD49d^+^ T cells (**Supplementary Figure 3**). Despite high proportions of T cells positive for *ITGAV*, we did not detect CD51 protein in any T-cell subsets in glioblastoma biopsies or paired blood samples (**Figures 5A, B**). Importantly, we demonstrated efficacy of this CD51 antibody clone with the Malme-3M human melanoma cell-line (data not shown). In contrast, CD49a (integrin α1) expression was significantly enriched within CD4^+^ Tcm, Tem, Temra and Tregs, and CD8^+^ Tcm, Tem and Temra subsets in glioblastoma infiltrates compared to blood, and both CD4^+^ and CD8^+^ Trm cells expressed this integrin (**Figures 5C, D**). Additionally, CD4^+^ Tcm, Tem and Tregs expressing CD49d (integrin α4) were enriched in glioblastoma (**Figure 5D**). High proportions of CD8^+^ T-cell subsets expressed CD49d, and enrichment for CD49d expression in glioblastoma was significant for the CD8^+^ Tcm population (**Figure 5D**). Given large proportions of T cells in blood and glioblastoma expressed CD11a and CD49d, the abundance of these integrins on T cells, as measured by gMFI, was compared between blood and glioblastoma samples (**Supplementary Figure 5**). We observed no differences in the abundance of CD11a for CD4^+^ T-cell subsets, while there was an increase in the abundance of CD11a on the CD8^+^ Temra population (**Supplementary Figures 5A, B**). There was a significant decrease in the abundance of CD49d on the CD4^+^ Tem population in glioblastoma compared to blood samples, and a significant increase in CD49d gMFI on CD4^+^ Tregs in glioblastoma (**Supplementary Figure 5A**). However, there was no difference in the abundance of CD49d on CD8^+^ T-cell subsets between glioblastoma and paired blood samples (**Supplementary Figure 5B**). Physiological T-cell surveillance of the brain includes migration across post-capillary blood-vessels into perivascular spaces adjacent to the brain parenchyma (12). This pathway requires adherence of T cells, which occurs *via* the integrins CD11a and CD49d interacting with adhesion molecules ICAM-1 and VCAM-1, respectively, on BBB ECs. Indeed, we identified expression of ICAM-1 and VCAM-1 at the gene level within glioblastoma-associated clusters *via* scRNA-seq (**Supplementary Figure 7A**). Together, these data suggest that CD11a-ICAM-1, CD49a and CD49d-VCAM-1 contribute to the infiltration of subsets of T cells into glioblastoma.

### Complementary Chemokines Are Detected in Glioblastoma Biopsies, CUSA Samples and Primary Glioblastoma Cell-Lines

**Figure 6:**
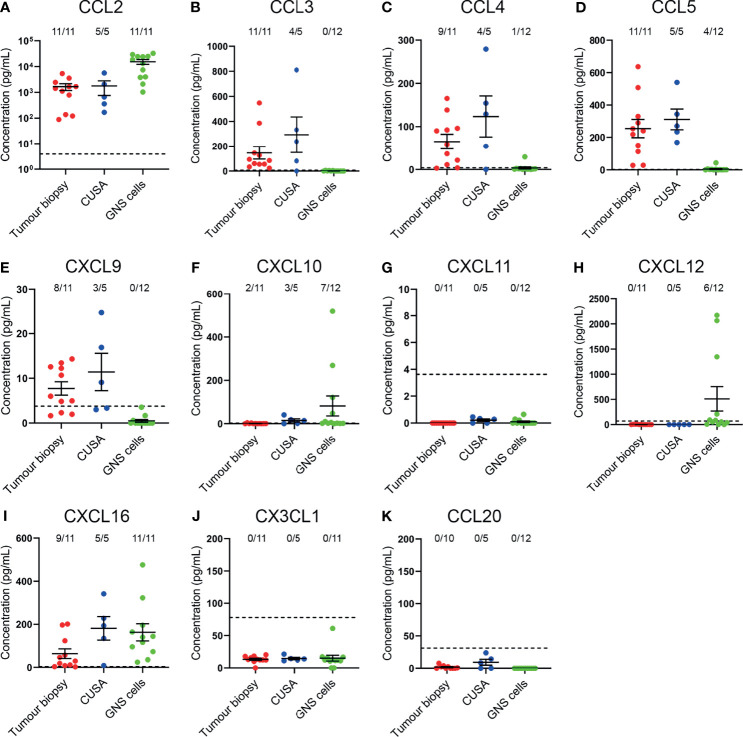
Complementary chemokines are detected in glioblastoma biopsies and CUSA samples, and supernatants from cultured GNS cells. **(A–K)** Chemokine abundance in supernatants from dissociated tumour biopsy, dissociated tumour fragments recovered from CUSA samples, and cultured GNS cells. Each dot represents one sample, bars represent mean ± SEM. Dotted line represents lower limit of detection. The number of samples above this threshold for each chemokine is displayed.

Having identified that CCR2^+^, CCR5^+^, CXCR3^+^, CXCR4^+^, and CXCR6^+^ T-cell subsets were enriched in glioblastoma, we subsequently validated the expression of complementary chemokines in dissociated glioblastoma biopsies and patient-derived glioblastoma cell-lines (**Figure 6**). We also tested for the complementary chemokines to CCR6 and CX3CR1 because these receptors have been implicated in brain-homing in other studies (28, 29). The CCR4 chemokines were not selected for further analysis as we observed low proportions of non-lymphocyte cell populations expressing the complementary chemokines CCL17 and CCL22 *via* scRNA-seq (**Figure 1G**) and observed no enrichment of CCR4^+^ T cells in glioblastoma (**Figures 3C, 4C**). To measure chemokines present within tumour tissue, the supernatant fraction from dissociated glioblastoma biopsies and CUSA aspirates, expected to contain soluble factors released from extracellular tissue spaces, was retained for analysis. In addition, we also collected supernatants from short-term cultures of tumour cells cultured under defined GNS cell conditions (24) to measure active secretion of chemokines by tumour cells. Chemokines were detected in both sample types *via* ELISA or cytometric bead-based assay. Notably, this approach favours the detection of soluble chemokines, as chemokines sequestered intracellularly or tethered to the cell surface *via* transmembrane domains or glycosaminoglycans (GAGs) are likely to remain in the cellular fraction. Of all chemokines assayed, the CCR2/CCR4 ligand CCL2 was the most abundant chemokine detected in tumour biopsy, CUSA and cultured GNS cell supernatants, and was present above the limit of detection in all samples analysed (**Figure 6A**). Interestingly, CCR5 ligands: CCL3, CCL4 and CCL5 were detected in the majority of supernatants harvested from dissociated tumour biopsy and CUSA, but little was detected in GNS cell supernatants (**Figures 6B–D**). Of the CXCR3 ligands, CXCL9 was detected in the majority of dissociated tumour specimens but was below the level of detection in all GNS cell cultures analysed (**Figure 6E**), CXCL10 was largely absent from dissociated tumour specimens, but highly expressed by few GNS cell samples (**Figure 6F**), and CXCL11 was below the lower limit of detection in all samples (**Figure 6G**). This was in contrast to expression of these ligands detected *via* scRNA seq (**Figure 1F**), but may reflect the high affinity of CXCL10 and CXCL11 for GAGs (30, 31). CXCL12, the sole chemokine ligand for CXCR4, was abundant in 6 out of 12 supernatants from the GNS cell cultures, however it was undetectable in all dissociated tumour biopsy and CUSA samples analysed (**Figure 6H**). CXCL16, the chemokine ligand of CXCR6, was detected in supernatants from the majority of dissociated tumour and GNS cell samples (**Figure 6I**). CX3CL1 was below the lower limit of detection in supernatants from all dissociated glioblastoma tumour biopsy, CUSA and GNS cell samples analysed (**Figure 6J**). However, measures here for both CXCL16 and CX3CL1 may underestimate their abundance as they are expressed as transmembrane chemokines and CXCL16 exhibits selective GAG binding (32–34). CCL20, the chemokine ligand of CCR6, was not detected in either dissociated tumour or GNS cell supernatants (**Figure 6K**). Comparing chemokine abundance between dissociated tumour and cultured GNS cell supernatants, CCL3, CCL4, CCL5 and CXCL9 were abundant in dissociated tumour samples, with little detected in supernatants from cultured tumour cells, suggesting non-tumour-cell populations were the source of these chemokines in glioblastoma. In contrast, glioblastoma tumour cells may be the main source of CCL2, CXCL10 and CXCL12 production in the tumour microenvironment given the elevated CCL2 detected in GNS cell culture supernatants and the minimal levels of CXCL10 and CXCL12 detected in dissociated tumour supernatants. Considered together with the analyses of receptor expression on glioblastoma T cells, these data implicate the CCR2-CCL2, CCR5-CCL3/4/5, CXCR3-CXCL9, CXCR4-CXCL12 and CXCR6-CXCL16 chemokine axes in T-cell infiltration and/or retention in glioblastoma.

## Discussion

Chemokine receptors and integrins play critical roles in chemotaxis, adhesion, and extravasation of T cells into tumours. It is widely reported that integrins expressed on T cells undergo conformational changes upon chemokine-chemokine receptor binding to facilitate greater adhesion to blood ECs (11, 35). Our work has identified potential chemokine-chemokine receptor axes and integrins that may direct T cells to glioblastoma. In our study, scRNA-seq analysis and protein validation implicate the CCR2-CCL2, CCR5-CCL3/4/5, CXCR3-CXCL9, CXCR4-CXCL12, and CXCR6-CXCL16 chemokine axes, and CD49a, CD49d, and CD11a integrins in glioblastoma infiltration. Few studies have investigated homing of T cells in glioblastoma, and our study is the first to systematically identify chemokine ligand and receptor pairs in primary tumour material from newly diagnosed glioblastoma patients. The brain is considered an immune-privileged site and migration of T cells to this tissue is normally tightly regulated. Whilst the BBB is compromised in glioblastoma, extravasation of T cells across the BBB is still a rate-limiting factor in successful CAR T-cell therapies (36). Therefore, understanding the factors that regulate entry of T cells into glioblastoma is important to tailor therapeutic approaches.

Our study is also the first to identify CXCR6^+^ T-cell subsets as being enriched in glioblastoma, as well as demonstrating CXCL16 expression in dissociated tumour biopsies and glioblastoma cell-lines. We identified CXCR6^+^ T cells enriched in CD4^+^ Tcm, Tem, Temra, and Tregs, and CD8^+^ Tcm and Temra populations. CXCR6, a marker of Trm cells, was indeed expressed by a high proportion of both CD4^+^ and CD8^+^ Trm cells in glioblastoma. The CXCR6-CXCL16 axis may regulate migration, retention or survival in glioblastoma. Indeed, this axis has been shown to be important for CD8^+^ T-cell anti-tumour responses or retention in tumours (19, 37) and critical for T-cell survival within murine melanoma tumours by positioning T cells with intratumoral dendritic cells (DCs) secreting pro-survival cytokines (38). Ultimately, the CXCR6-CXCL16 axis has been demonstrated to be essential in CD8^+^ T-cell responses to ICI therapy and mediating cancer regression in mouse models of colorectal cancer and melanoma (39). Thus, CAR T cells engineered to express CXCR6 exerted superior anti-tumour activity in pre-clinical models of pancreatic cancer compared to CAR T cells without this genetic modification (40). Whether CXCR6 drives T-cell migration, retention or survival in glioblastoma remains an important question given its requirement in CAR T-cell and ICI therapy in other tumour models.

Importantly, we have shown that CCR5^+^ cells are significantly enriched in all glioblastoma-infiltrating T-cell subsets, and CCR5 ligands were detected in supernatants from dissociated glioblastoma biopsies, implicating a key role for this axis in the infiltration and/or retention of T cells in glioblastoma. Enrichment of CCR5^+^ T cells has been observed in other cancer types, suggesting a potential role for migration to these tumours (41). However, whether CCR5 mediates migration to the brain and glioblastoma remains unclear. Higher proportions of CCR5^+^ T cells have been identified in human cerebrospinal fluid (CSF) compared to peripheral blood (42) and effector CCR5^+^CD8^+^ T cells were found to be enriched in human glioblastoma (43). Therefore, the importance of the CCR5 chemokine receptor axis in glioblastoma infiltration should be explored further.

The CXCR4-CXCL12 axis has known roles in T-cell surveillance and infiltration of the brain parenchyma during homeostasis and central nervous system (CNS) pathologies (44, 45). Here, we observed high proportions of both patient blood and glioblastoma T-cell subsets expressing CXCR4, and significantly greater abundance of CXCR4, by measure of gMFI, on glioblastoma T-cell subsets. However, in contrast to enrichment of CXCR4^+^ T cells in glioblastoma, the greater abundance of CXCR4 on these cells may reflect redundancy of this axis in tumour infiltration as ligation of CXCR4 by CXCL12 results in receptor internalisation and desensitisation (46). We were unable to detect CXCL12 in supernatants from dissociated glioblastoma tumour biopsy and CUSA samples. This may be attributed to the intracellular or membrane-bound localisation of CXCL12 observed in tumour-associated ECs (47). Interestingly, in mouse models of multiple sclerosis, T cells that have passed BBB-associated ECs are restricted to the perivascular niche by CXCL12 localised to the basolateral surface of these ECs (45). Thus in multiple sclerosis, CXCR4 restricts T cells migrating across astrocyte endfeet and entering the brain. Whether similar brain infiltration mechanisms are employed in glioblastoma remain unknown. However, CXCR4-blockade in combination with anti-PD-1 has been shown to have a synergistic effect in increasing the proportion of IFNγ^+^ and TNFα^+^ producing CD4^+^ and CD8^+^ T cells in the brains of mice with glioblastoma (48).

The CCR2/CCR4-CCL2 axis has been identified in previous *in vitro* glioblastoma studies. High abundance of CCL2 has been detected in glioma tumour tissue and cell-line supernatants by ELISA, and glioblastoma-patient peripheral blood Tregs could be recruited to glioblastoma-conditioned medium in a CCL2-dependent manner (49, 50). We also observed high levels of CCL2 in supernatants from dissociated glioblastoma biopsies and GNS cells, inferring that CCL2 could recruit T cells through CCR2 or CCR4 to glioblastoma. However, here we show that T cells are more likely to be recruited *via* CCR2 than CCR4 given that both CCR2^+^ Tregs, and CCR2^+^ Tcm cells were enriched in glioblastoma tissues, and we found no enrichment of CCR4^+^ T cells in glioblastoma. Indeed, pathogenic Th17 cells have been shown to migrate to the inflamed CNS in murine models of multiple sclerosis *via* CCR2 (51). Thus, our findings now suggest that high CCL2 abundance identified by multiple glioblastoma studies may mediate trafficking to glioblastoma through CCR2 rather than CCR4. This remains an important question for future studies.

CXCR3 is widely expressed by activated T cells and was highly enriched on glioblastoma-infiltrating T-cell subsets. Studies investigating brain viral infection have demonstrated that CXCR3 mediates T-cell entry from the CSF into the brain parenchyma (52, 53). Additionally, T cells in human CSF are enriched for CXCR3^+^ and CCR5^+^ compared to peripheral blood (42). A study investigating the role of CXCR3 signalling in glioblastoma observed that CXCR3-KO mice had a reduced survival compared to WT mice (54). However, glioblastoma-infiltrating T-cell numbers were not significantly different between groups, suggesting that CXCR3 did not mediate infiltration of T cells in this model. In keeping with this, recent studies in subcutaneous mouse tumour models have shown that CXCR3 is not required for tumour infiltration but is important for positioning T cells to receive anti-tumour activation signals from intratumoral DCs when paired with anti-PD-1 therapy (55). Therefore, these discrepancies highlight the need for future studies using immunocompetent mouse glioblastoma models as it is unclear whether CXCR3 mediates infiltration or positioning of T cells within glioblastoma. Identifying the function of CXCR3 will better instruct the use of immunotherapies to treat glioblastoma *via* augmenting CXCR3 expression on CAR T cells infused *via* the CSF to improve entry to the brain, or improve T cell-DC interactions to enhance anti-tumour responses when paired with ICI therapy.

Despite high proportions of T cells and non-lymphocyte populations identified as positive for *CCR6* and *CCL20 via* scRNA-seq respectively, CCR6^+^ T cells were not enriched in glioblastoma and we were unable to detect CCL20 in supernatants *via* ELISA. A prior study has identified CCL20 expression by immunohistochemistry within human glioblastoma tissues, and this has correlated with increased T-cell infiltration (56). Notably however, CCL20 was limited to the cytoplasm of tumour cells, with little secreted in the glioblastoma microenvironment. This may explain why we did not detect CCL20 by ELISA in supernatants from dissociated glioblastoma specimens or GNS cells. Additional work is necessary to determine whether the high proportion of cells expressing *CCL20* has any biological significance given our data showed a lack of enrichment of CCR6^+^ T-cell subsets and no detection of CCL20 protein by ELISA.

CX3CL1 is constitutively expressed in the healthy brain by neurons and astrocytes, and exists in transmembrane and soluble forms (32, 57). Considering that only the soluble form would be present in supernatants, this may explain why CX3CL1 protein was below the limit of detection for all glioblastoma biopsies and GNS cell-lines analysed despite varying proportions of cells expressing *CX3CL1* identified by scRNA-seq analysis. Regardless, a study using a murine model of glioma found no expression of *Cx3cr1* in glioma-infiltrating T cells, nor a role for this receptor in T-cell recruitment to glioma (58). Similarly, we observed a decrease in the proportion of CX3CR1^+^CD4^+^ Temra and CX3CR1^+^CD8^+^ Tem and Temra subsets in glioblastoma compared to paired blood samples, suggesting little requirement for CX3CR1 in glioblastoma infiltration.

We found high expression of CD11a^+^, and enrichment of CD49a^+^ and CD49d^+^ T cells in glioblastoma. We also identified expression of ICAM-1 and VCAM-1, the cognate receptors for the CD11a and CD49d integrins respectively, at the gene level within glioblastoma-associated clusters *via* scRNA-seq. CD11a and CD49d have been implicated in T-cell glioblastoma-infiltration in a previous study *in vitro*, where transmigration of T-cells across glioblastoma-isolated ECs occurred in a ICAM-1 and VCAM-1-dependent manner (59). Furthermore, neutralising CD49d *in vivo* abrogates homing of cytotoxic T cells to intracranial tumours (60). In line with this, we found high frequencies of all T-cell subsets expressing CD49d in glioblastoma tissues and uniformly high expression of CD11a. Unlike CD11a and CD49d, a role for CD49a in glioblastoma has not been investigated. CD49a is a type IV collagen receptor, a marker of T-cell tissue residency, and CD49a^+^ Trm cells have been observed to populate the human brain (18). Accordingly, the greatest frequency of CD49a^+^ cells identified here were the Trm populations, yet moderate proportions of multiple CD4^+^ and CD8^+^ T-cell subsets expressed CD49a and were enriched in glioblastoma. Early studies in other tumour models have demonstrated CD49a expression is critical for CD8^+^ T cell anti-tumour responses at mucosal sites (61). Importantly, a recent study investigating the functionality of CD49a in melanoma tumour models has shown that CD49a is upregulated on tumour-infiltrating T cells by the tumour microenvironment and enhances T-cell motility (62). Given the likely function of CD49a in tumour retention, its role in glioblastoma should be investigated further.

A limitation of our study is that enrichment of migratory receptors within tumours does not demonstrate their functional requirement. Here, enrichment of migratory receptors within glioblastoma suggests that they may be important in the processes underlying tumour infiltration, or receptor expression may be induced in T cells by the tumour environment following infiltration. Alternatively, the enrichment of chemokine receptors might be a bystander effect of memory or effector T cells concentrating at the tumour site, and not as a result of their requirement for tumour infiltration. Furthermore, additional modifications and downstream signalling molecules will also affect chemokine receptor responsiveness and activity (63). However, having identified key receptors amongst all known homing receptors, further functional studies with orthotopic xenograft glioblastoma tumour models and syngeneic immunocompetent models can identify receptor-dependent roles in glioblastoma-homing. These models would directly address the requirement of these receptors in glioblastoma infiltration and identify receptors with non-chemotactic or redundant functions in anti-tumour responses. Importantly, investigating the endogenous intratumoral T-cell migratory profile is an approach that can be leveraged to identify unique migratory receptors important for T-cell infiltration in other solid tumours and guide subsequent functional assays. In turn, by enhancing the expression of appropriate migratory receptors, T-cell-based therapies could be improved and implemented in clinical trials for glioblastoma and possibly other solid tumours.

## Data Availability Statement

The raw sequencing datasets presented in this article are not readily available due to patient confidentiality, as required by our Human Research Ethics Committee. The processed gene expression matrix with raw mRNA counts for all cells in the T cell cluster has been made publicly available. This data can be found on figshare: 10.6084/m9.figshare.19119698. Other original datasets presented in the study are either contained in the article or will be made available by the authors, *via* the corresponding author (TG, tessa.gargett@sa.gov.au).

## Ethics Statement

The studies involving human participants were reviewed and approved by Central Adelaide Local Health Network Human Research Ethics. The patients/participants provided their written informed consent to participate in this study.

## Author Contributions

PK: Conceptualisation, data curation, formal analysis, investigation, methodology, writing – original draft, writing – review & editing. LE:Conceptualisation, data curation, investigation, funding acquisition, methodology, resources, supervision, writing – review & editing. JT: Data curation, formal analysis, methodology, writing – review & editing. CB: Conceptualisation, data curation, methodology, writing – review & editing. RO: Resources. SIP: Resources. SL:Resources. MT: Resources. SMP: Funding acquisition, resources. GG: Funding acquisition, resources, writing – review & editing. MB: Conceptualisation, funding acquisition, resources, supervision, writing – review & editing. TG: Conceptualisation, funding acquisition, methodology, resources, supervision, writing – review & editing. All authors contributed to the article and approved the submitted version.

## Funding

This work was supported by the Neurosurgical Research Foundation, The Hospital Research Foundation Group, a Tour de Cure Senior Research Grant, the Cancer Council SA Beat Cancer Project, the Royal Adelaide Hospital Research Fund, and the Health Services Charitable Gifts Board (Adelaide). scRNA-seq experiments were funded by a Cure Brain Cancer Foundation Infrastructure Grant to SMP, GG, and MB. SMP was supported by the National Health and Medical Research Council of Australia (1156693). GG was supported by the Australian Research Council (FT16010036). PK was supported by a University of Adelaide Honours Scholarship.

## Conflict of Interest

The authors declare that the research was conducted in the absence of any commercial or financial relationships that could be construed as a potential conflict of interest.

## Publisher’s Note

All claims expressed in this article are solely those of the authors and do not necessarily represent those of their affiliated organizations, or those of the publisher, the editors and the reviewers. Any product that may be evaluated in this article, or claim that may be made by its manufacturer, is not guaranteed or endorsed by the publisher.
