# Supplementary material for: Characterising Distinct Migratory Profiles of Infiltrating T-Cell Subsets in Human Glioblastoma
Source: Front Immunol. 2022 Apr 6;13:850226. doi: 10.3389/fimmu.2022.850226 (PMC9019231; doi:10.3389/fimmu.2022.850226)
Supplement: Supplementary file 1 [file DataSheet_1.docx]

Supplementary Material


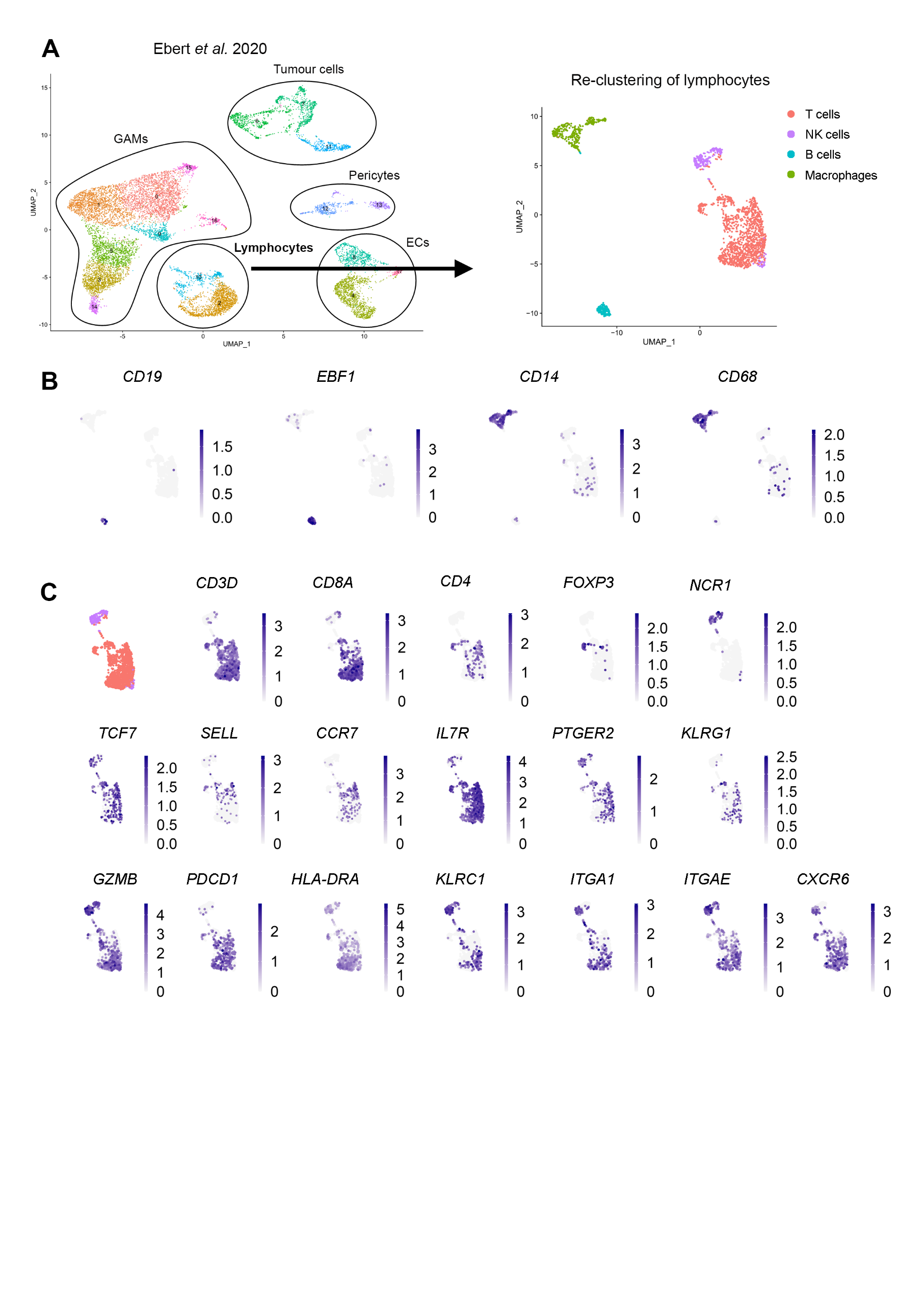


**Supplementary Figure 1. Bioinformatics strategy used to identify T-cell populations in lymphocyte cluster. (A)** Lymphocyte clusters were removed from non-lymphocyte populations and re-clustered to segregate T cells from non-T-cell lymphocyte populations based on differential expression of genes (adapted from Ebert et al., 2020). The lymphocyte UMAP plot identifies T cells, NK cells, B cells and contaminating macrophages upon re-clustering of the original lymphocyte cluster. **(B, C)** UMAP plots, where the colour gradient corresponds to the level of gene expression within each cell, were used to identify lymphocyte and T-cell populations by visualising expression of lineage-markers. **(B)** B-cell and contaminating macrophage clusters were identified by visualising expression of the canonical markers *CD19* and *EBF1*, and *CD14* and *CD68* respectively. **(C)** CD8^+^ and CD4^+^ T cells, Tregs and NK cells were identified by visualising *CD3D*, *CD8A*, *CD4*, *FOXP3*, and *NCR1* genes respectively. Memory (*TCF7*, *SELL*, *CCR7*, *IL7R*, *PTGER2*, *KLRG1*) and effector (*GZMB*, *PDCD1*, *HLA-DRA*, *KLRC1*) genes were visualized to identify possible Tcm- and Tem-phenotypes of sequenced glioblastoma T cells. *ITGA1, ITGAE* and *CXCR6* were used to identify T cells with a Trm-phenotype. **(A-C)** n=3 patient glioblastoma biopsies.


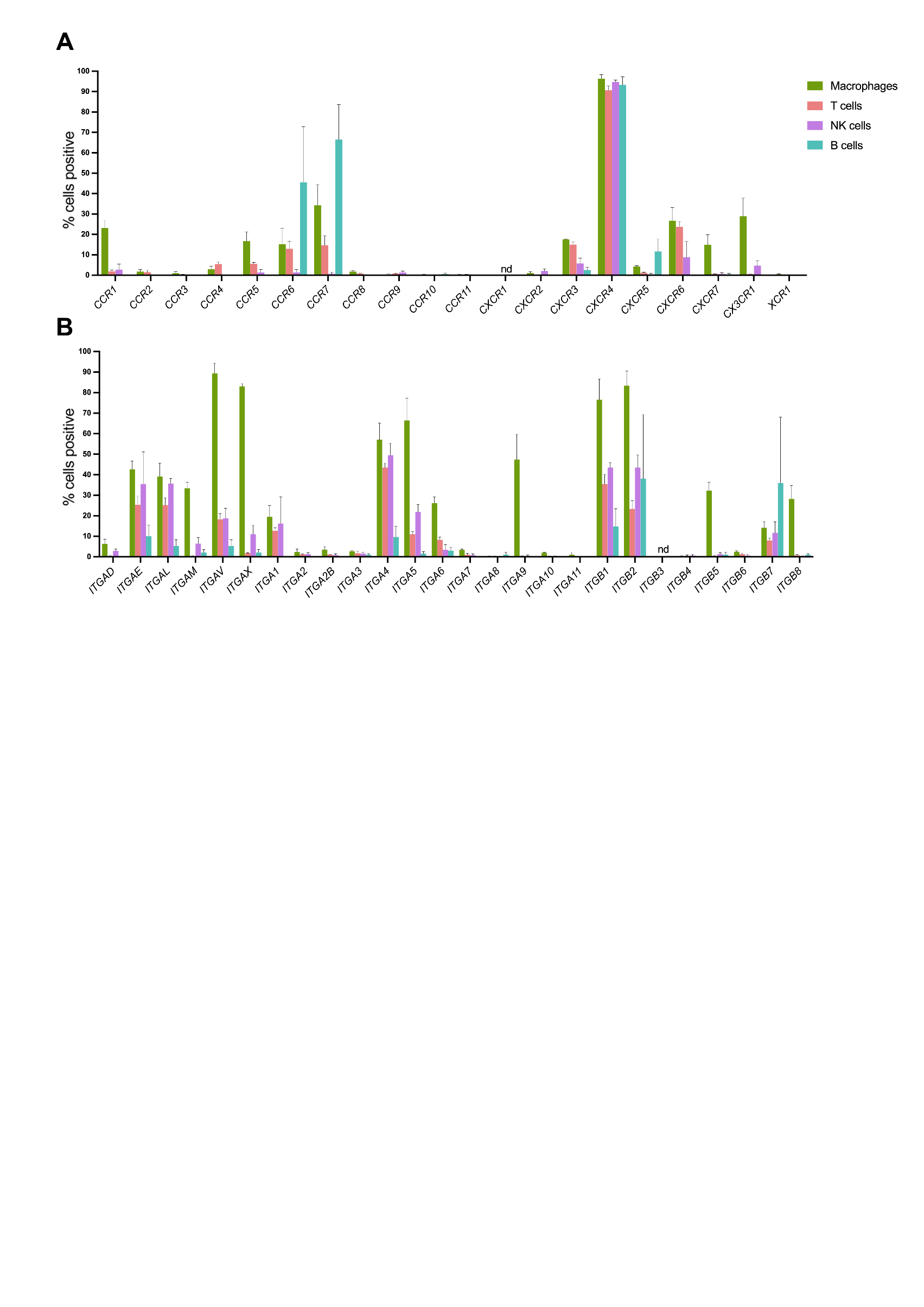


**Supplementary Figure 2. Lymphocyte cluster populations express distinct chemokine receptor and integrin genes.** Frequency of macrophages, T cells, NK cells and B cells positive for **(A)** chemokine receptor and **(B)** integrin genes. Bars represent mean ± SEM. n=3 patient glioblastoma biopsies.


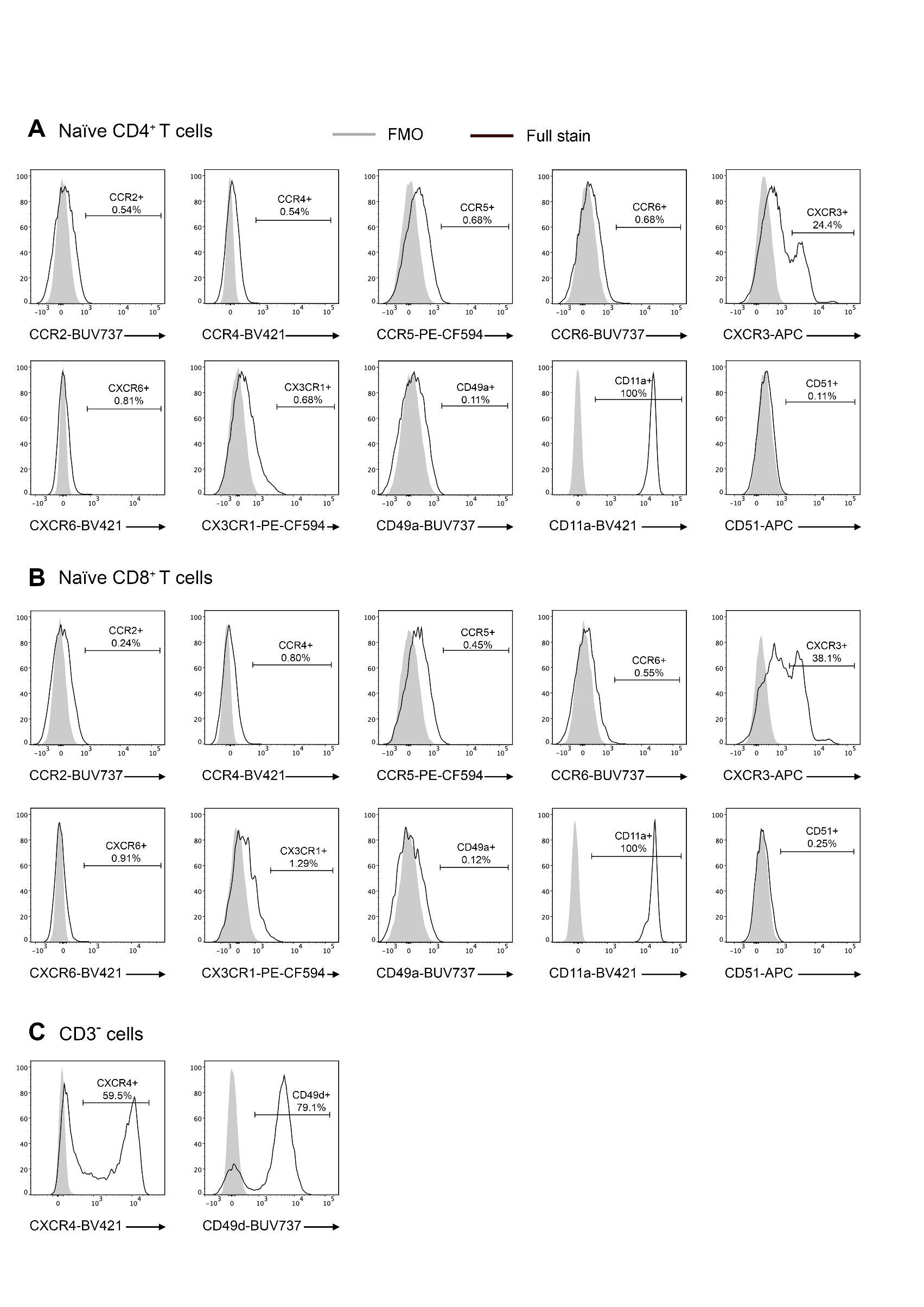


**Supplementary Figure 3. Naïve T cells and CD3^-^ cells in peripheral blood serve as internal negative controls for chemokine receptor and integrin expression.** Representative histograms showing chemokine receptor and integrin expression in **(A)** naïve CD4^+^, **(B)** naïve CD8^+^ T cells and **(C)** CD3^-^ cells (black line) compared to fluorescence minus one (FMO) controls (grey filled) in healthy peripheral blood samples. **(A, B)** Naïve CD4^+^ and CD8^+^ T cells lack expression of most receptors except for CXCR3 and CD11a, where peaks corresponding to positive populations were used to set gates. **(C)** CD3^-^ cells, which contained distinct positive and negative populations of CXCR4 and CD49d, were used to set positive gates for these receptors. Plots are representative of healthy peripheral blood samples n=3.


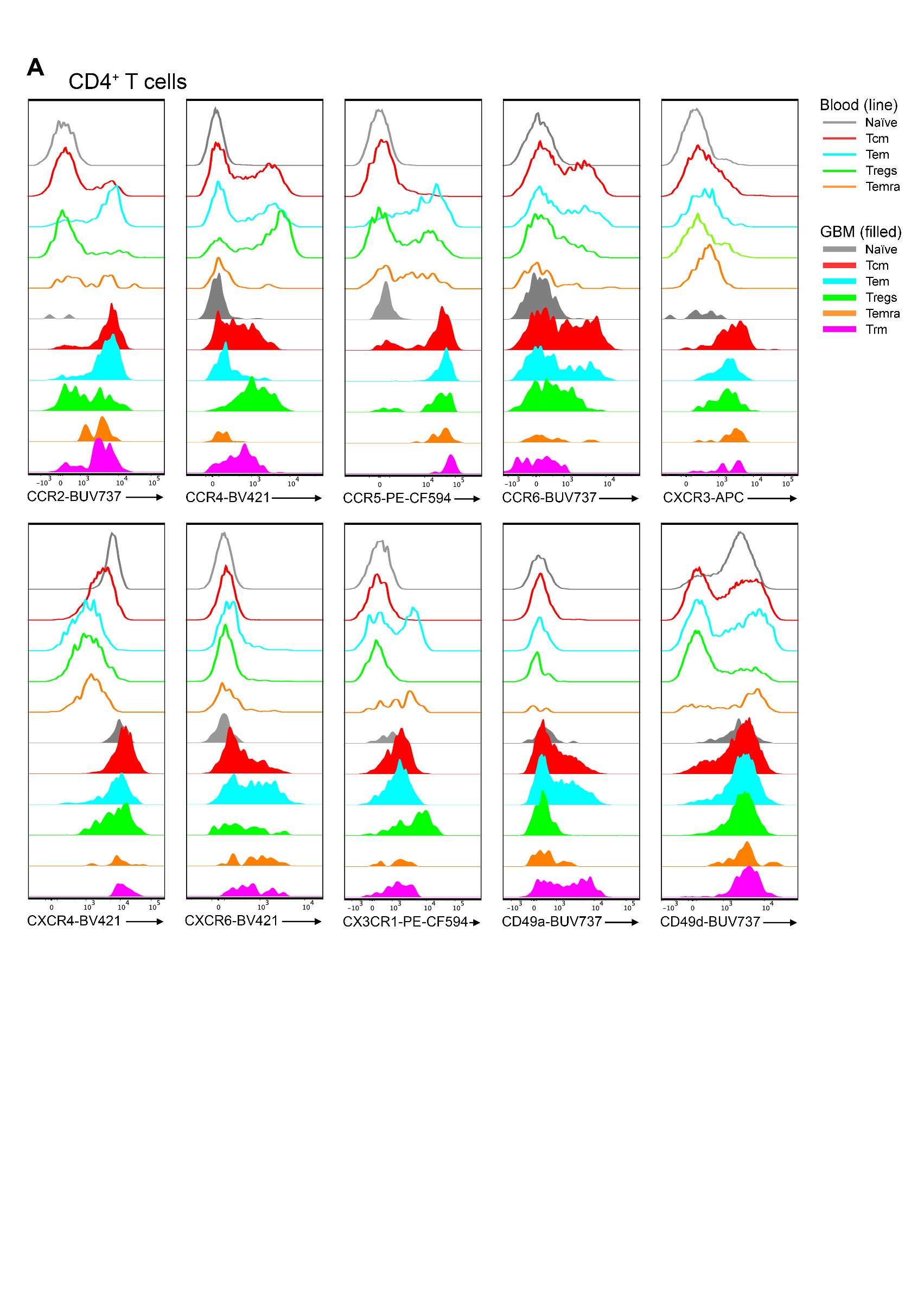


**Supplementary Figure 4. Chemokine receptor and integrin expression on paired blood and glioblastoma CD4^+^ T-cell subsets. (A)** Representative histograms showing chemokine receptor and integrin expression within CD4^+^ T-cell subsets in glioblastoma (GBM) (filled) and paired blood samples (line). Representative of matched blood and GBM samples n=5-8 each.


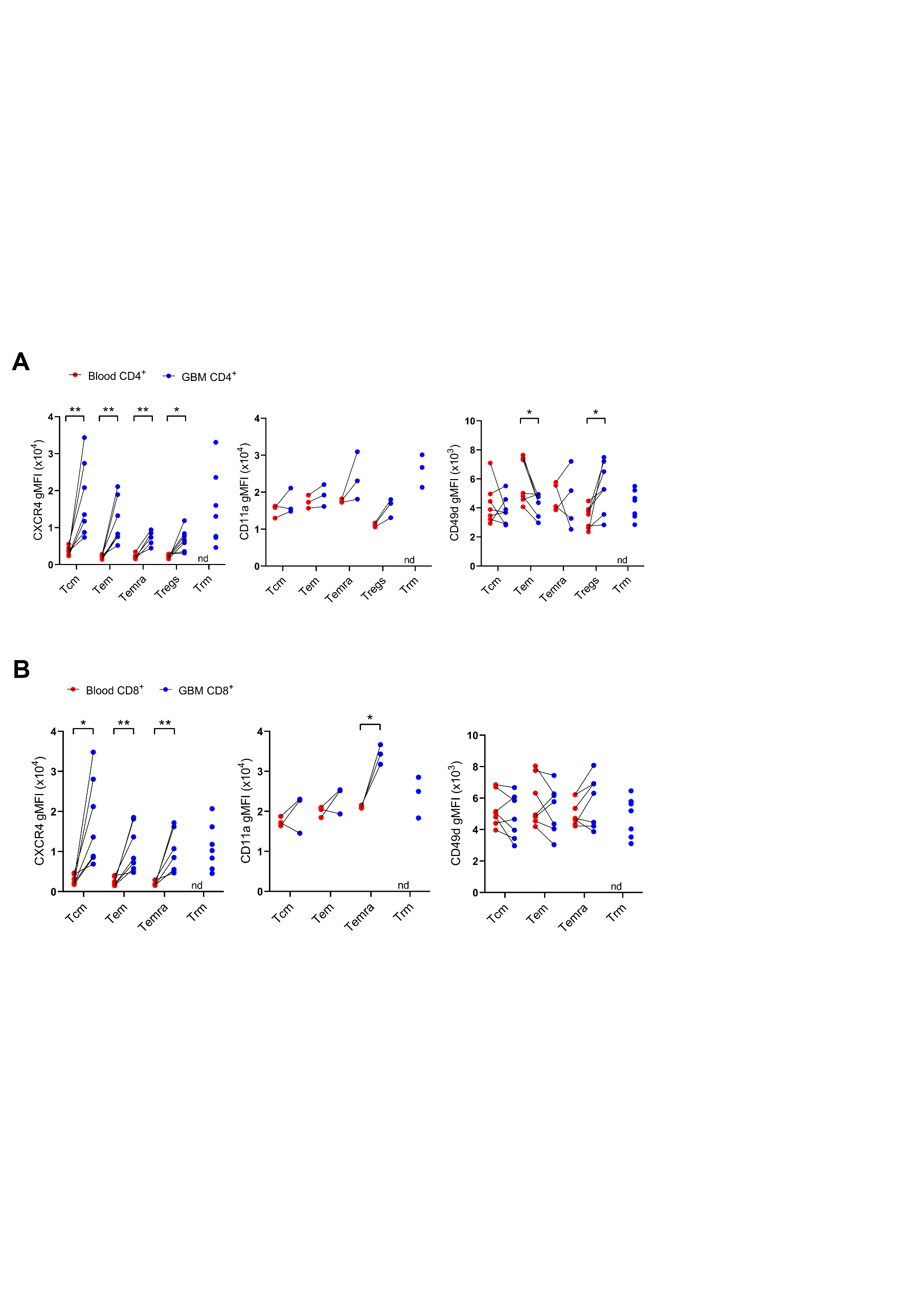


**Supplementary Figure 5. Abundance of CXCR4, CD11a and CD49d on glioblastoma T-cell subsets compared to paired blood samples. (A, B)** Geometric mean fluorescence intensity (gMFI) of CXCR4, CD11a and CD49d on **(A)** CD4^+^ and **(B)** CD8^+^ T-cell subsets in blood and matched glioblastoma (GBM) biopsies. The gMFI was calculated within the positive gates for each receptor. Each dot represents one patient, lines represent paired patient samples. Two-tailed paired Student’s t test, *p<0.05, **p< 0.01, nd=not detected.


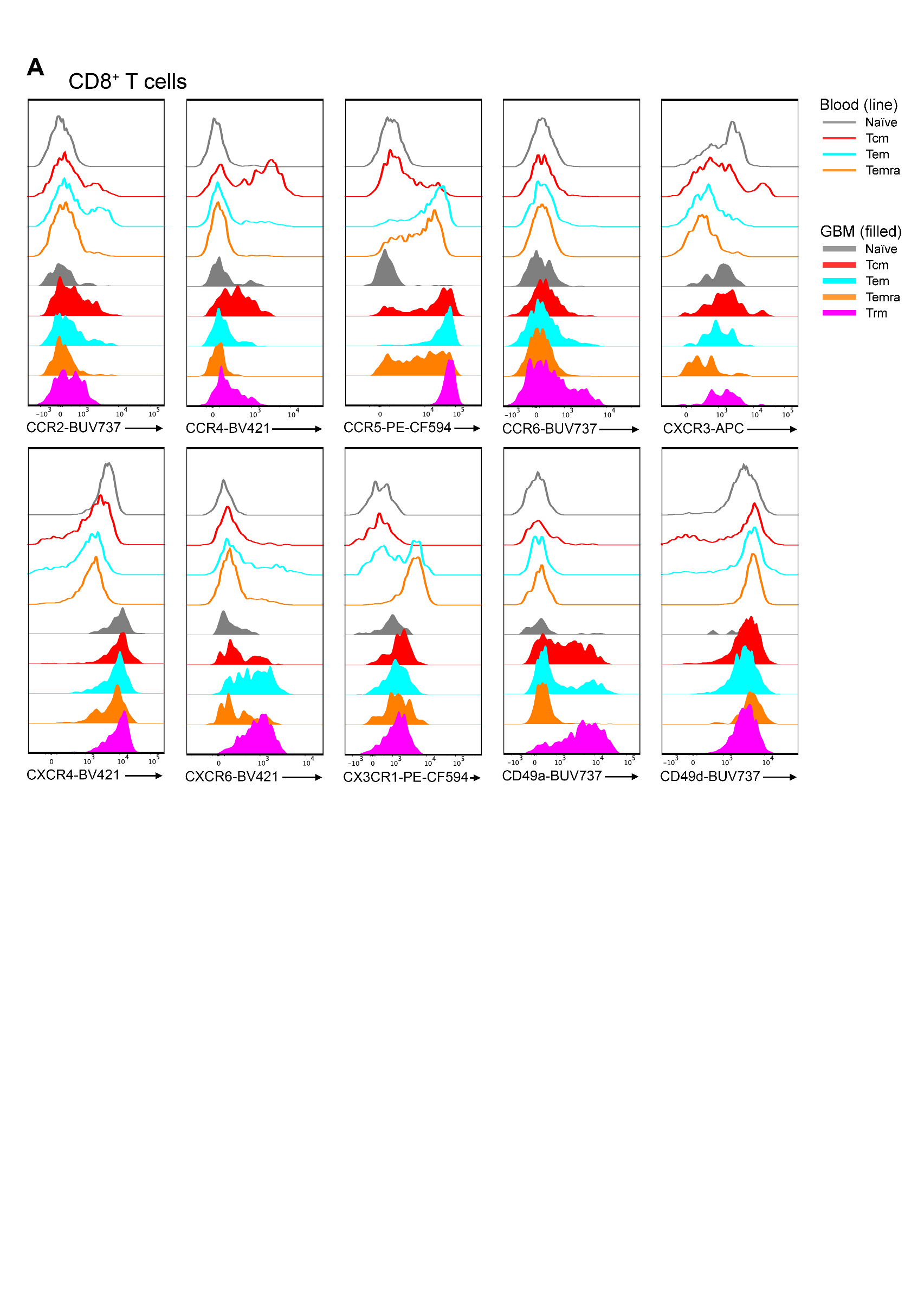


**Supplementary Figure 6. Chemokine receptor and integrin expression on paired blood and glioblastoma CD8^+^ T-cell subsets. (A)** Representative histograms showing chemokine receptor and integrin expression within CD8^+^ T-cell subsets in glioblastoma (GBM) (filled) and paired blood samples (line). Representative of matched blood and GBM samples n=5-8 each.


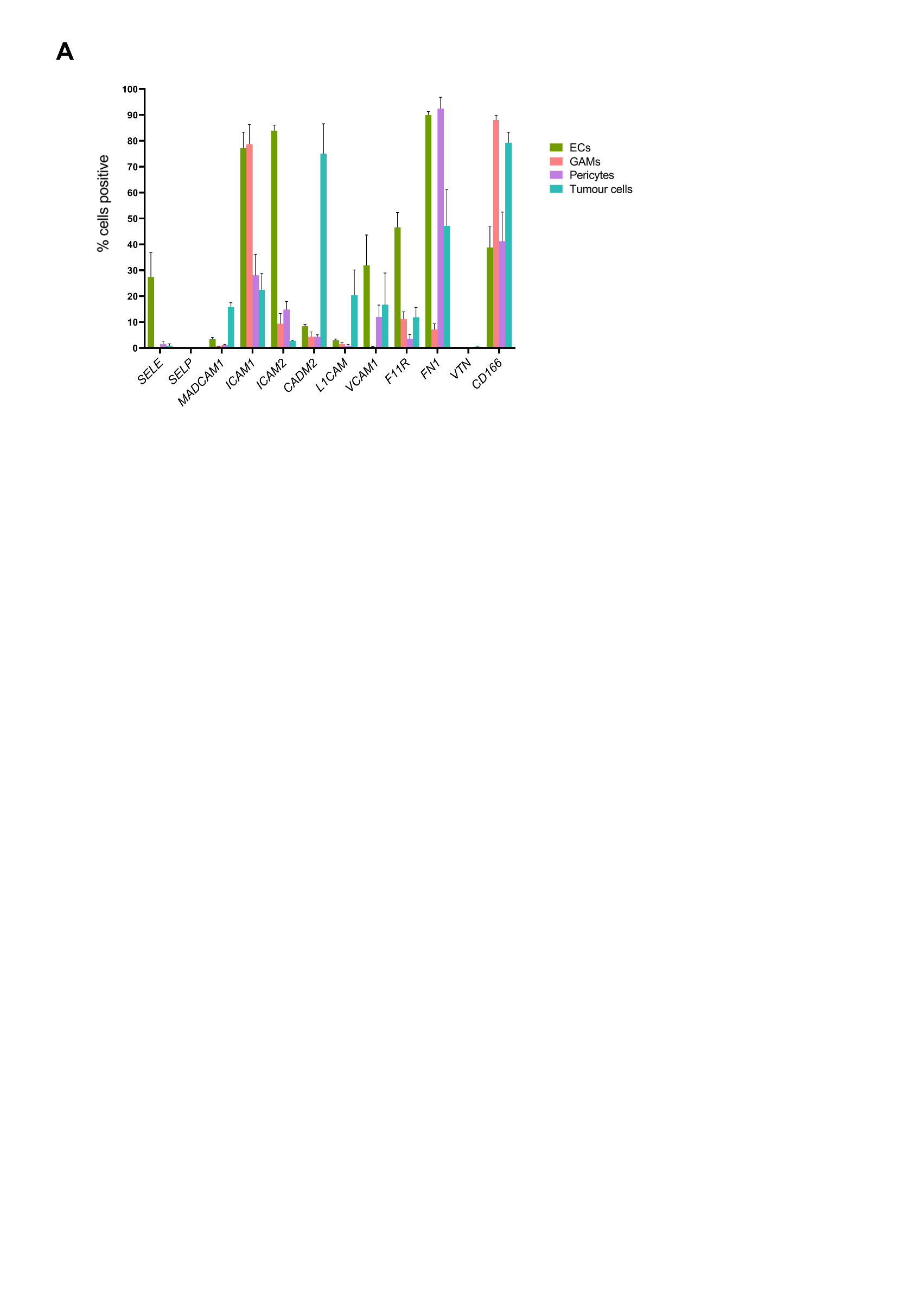


**Supplementary Figure 7. Glioblastoma tumour cells and tumour-associated cell populations express distinct adhesion molecules genes.** **(A)** Frequency of endothelial cells (ECs), glioblastoma-associated macrophages/microglia (GAMs), pericytes and tumour cells positive for adhesion molecule genes. Bars represent mean ± SEM. n=3 patient glioblastoma biopsies.
